# Data-driven simulations for training AI-based segmentation of neutron images

**DOI:** 10.1038/s41598-024-56409-3

**Published:** 2024-03-19

**Authors:** Pushkar S. Sathe, Caitlyn M. Wolf, Youngju Kim, Sarah M. Robinson, M. Cyrus Daugherty, Ryan P. Murphy, Jacob M. LaManna, Michael G. Huber, David L. Jacobson, Paul A. Kienzle, Katie M. Weigandt, Nikolai N. Klimov, Daniel S. Hussey, Peter Bajcsy

**Affiliations:** 1 Information Technology Laboratory, NIST, Gaithersburg, MD 20899 USA; 2 NIST Center for Neutron Research, Gaithersburg, MD 20899 USA; 3 Physical Measurement Laboratory, Gaithersburg, MD 20899 USA; 4 Department of Chemistry and Biochemistry, University of Maryland, College Park, MD 20742 USA

**Keywords:** INFER, Neutron imaging, Data-driven simulation, Semantic segmentation, Characterization and analytical techniques, Imaging techniques, Techniques and instrumentation, Techniques and instrumentation

## Abstract

Neutron interferometry uniquely combines neutron imaging and scattering methods to enable characterization of multiple length scales from 1 nm to 10 µm. However, building, operating, and using such neutron imaging instruments poses constraints on the acquisition time and on the number of measured images per sample. Experiment time-constraints yield small quantities of measured images that are insufficient for automating image analyses using supervised artificial intelligence (AI) models. One approach alleviates this problem by supplementing annotated measured images with synthetic images. To this end, we create a data-driven simulation framework that supplements training data beyond typical data-driven augmentations by leveraging statistical intensity models, such as the Johnson family of probability density functions (PDFs). We follow the simulation framework steps for an image segmentation task including Estimate PDFs $\,\rightarrow \,$ Validate PDFs $\,\rightarrow \,$ Design Image Masks $\,\rightarrow \,$ Generate Intensities $\,\rightarrow \,$ Train AI Model for Segmentation. Our goal is to minimize the manual labor needed to execute the steps and maximize our confidence in simulations and segmentation accuracy. We report results for a set of nine known materials (calibration phantoms) that were imaged using a neutron interferometer acquiring four-dimensional images and segmented by AI models trained with synthetic and measured images and their masks.

## Introduction

Neutron imaging^1^ (NI), small-angle neutron scattering^2,3^ (SANS) and ultra-small-angle neutron scattering^4^ (USANS) modalities are used respectively for micro and meso-scale material characterization. Neutron interferometric microscopy of small forces and hierarchical structures (INFER) uses 2-grating interferometry to combine the benefits of both modalities^5,6^. With INFER, the autocorrelation length is varied by changing the moire period and wavelength while maintaining a constant sample-detector distance. This is different than conventional Talbot-Lau interferometers (TLIs) which have a fixed period and operating wavelength. TLIs have a lower range of a few 10 nm, whereas in the INFER project we expect 1 nm to be the low range and 10 µm the high range, without a varying geometric blur. This range of autocorrelation lengths is possible because the 2-grating far field interferometer allows one to vary the moire period by varying the separation of the two phase gratings. There is significant visibility (high contrast) over several orders of magnitude in grating separation^7^.

NIST has been working on a prototype instrument that is capable of measuring this range of scales. The applications of multi-scale hierarchical characterization by INFER are wide-ranging, for example, measuring samples of civil engineering structures (e.g., cement^8^), polymers^9^, additive manufacturing^10,11^, steels^12^ and magnetic domains^13^, chemistry of complex systems (e.g., batteries^14,15^ and fuel cells^16,17^), and complex hierarchical structures (e.g., wood^18^ and bones^19^) as well as in geology^20^ and food science^21^.

Building, operating, and using neutron interferometers requires large amounts of resources, which imposes constraints on the acquisition time and on the number of measured images per sample. Segmentation of these measured images is valuable for performing automated and enhanced multi-modal analysis, understanding complex systems, as well as improving modeling and simulation. However, a very small number of measured images is insufficient for the training of supervised artificial intelligence (AI) models. While scientists are interested in measuring many samples, segmentation of measured images by hand becomes very time consuming and almost labor prohibitive. These constraints on measurements and analyses introduce a trade-off between the number of measured images per sample and the cost of each measurement associated with the instrument time in addition to manual segmentation labor. The motivation of our work is to minimize the cost associated with the manual segmentation labor.

The measured four-dimensional (4D) INFER data consist of spatial *x* and *y*, autocorrelation length ($\xi$), and attenuation (H0) and 1st imaging mode (H1) dimensions defined according to the nomenclature in H. Wen^22^. Dark-Field (DF), which is valuable in understanding microstructure of samples, is derived from H0 and H1 (see Section “Materials and data” for more information). For a measured (4D) INFER image collection, our objective is to automate an image segmentation task into accurate and semantically meaningful 2D regions along the x- and y-dimensions, where the region labels correspond to material types. In addition, the segmentation method should (ideally) generalize to images of other samples and computationally scale to the throughput of the INFER instrument (about 2 terabytes (TB) of tomographic projections per day).

**Figure 1:**
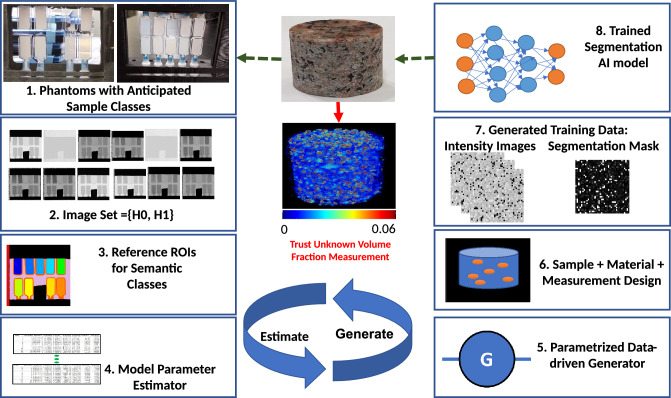
Overall approach for training AI models (1) Phantom (reference) materials are prepared. (2) INFER images of phantom are acquired. (3) Reference ROIs are annotated in a mask image. (4, 5) Model parameters are estimated and regenerated for validation. (5, 6) Scene masks are designed and populated with intensities $\hbox {generated}^{1}$ following the data-driven model. (7) Sets of designed masks and corresponding generated intensity images are split for training and validating an AI segmentation model. (8) AI models are trained on these datasets.

Our approach is to use supervised AI models trained on data-driven synthetic images for image segmentation. The experimental and computational workflow consists of the steps illustrated in Fig. 1. First, phantom (reference) materials are prepared, imaged using the INFER instrument, and geometrically corrected and normalized (steps 1 and 2). Next, the measured images are manually segmented into regions of interest (ROIs) that correspond to unique materials (step 3). From measured images and their masks, data-driven model parameters are estimated per ROI in step 4 and re-generated for statistical validation in step 5. In step 6, a set of masks (containing ROIs with unique labels) is generated and then populated with intensity values according to the extracted data-driven model per label (step 7). Finally, the intensity images and masks are used for training an AI segmentation model by presenting intensity images as inputs and masks as outputs. The methodology is validated by applying the trained AI model to measured images and evaluating accuracy of segmented ROIs against the manually created mask in step 3. This can then be iteratively tested against an unknown sample (e.g. granite block^20^).

The novelty of this work is in leveraging the Johnson family of PDFs in designing a methodology based on the following steps: Estimate PDFs $\,\rightarrow \,$ Validate PDFs $\,\rightarrow \,$ Design Image Masks $\,\rightarrow \,$ Generate Intensities $\,\rightarrow \,$ Train AI Model for Image Segmentation. The contributions lie in utilizing a statistical data-driven model for neutron beam imaging, designing simple scene masks for training segmentation models, and training AI segmentation models for neutron imaging experiments using the data-driven simulations.

This paper is organized as follows. Section “Related work” presents related work to each component of our overall approach shown in Fig. 1. Section “Experimental results” contains the experimental results. The results are discussed in Section “Discussion” and conclusions can be found in Section “Conclusions and future work”. Section “Methods” outlines the details of the methodology. In addition, Supplementary sections include Figures, Tables, and Discussion related to the many facets of the data-driven simulation approach.

## Related work

We divided related work into image simulations and AI model training because the scope of our work is based on novel image simulation model and on automating an image segmentation task by a trained AI model with annotated intensity images. Due to the limited measured data, we explore simulation methods for creating intensity images and annotation masks, validating the quality of simulated images, manually annotating measured images, and integrating simulated and measured data into a training dataset for AI models.

### Image simulations

Each pixel of the INFER images extracted along the autocorrelation length dimension produces a correlogram, which is related to small-angle scattering through a Hankel transform and can be considered a real space version of a SANS curve. With the billions of pixels to be analyzed per experiment, it is infeasible to efficiently analyze the data using manual inspection. At the same time, this amount of training data remains insufficient to train a supervised segmentation model to accurately automate the data analysis.

#### Methods for expanding image datasets for AI training

The problem of insufficient training data has been addressed in the past by using (a) augmentation techniques, (b) generative adversarial networks (GANs), and (c) transfer learning from pre-trained AI models^23^. Augmentation methods have been surveyed in a review^24^ as they have shown success in training deep learning algorithms and have support in the Albumentations Python library^25^. In the case of INFER data, the low signal-to-noise ratio (SNR) poses a significant challenge to the effective application of augmentation methods. GANs are known to suffer from instability (i.e., optimizing the min-max cost function) and dependency on model initialization^26^.

The success of transfer learning techniques depends on the overlap of image characteristics between the images used for a pre-trained AI model and INFER images. It has been demonstrated experimentally that the overlap between Common Objects in Context (COCO)^27^ images used in a pre-trained AI model and an INFER image is minimal^28^. Scientific data such as INFER images often are different in composition and nature to the COCO dataset. Thus, transfer learning is not expected to be beneficial when using pretrained models. Based on these considerations, our work is pursuing the option of expanding small training datasets using scene simulations.

#### Simulation methods

Simulation methods can be divided into physics-based and data-driven methods. There exist several physics-based simulation models for already well-established neutron scattering instruments, such as spin echo small-angle neutron scattering (SESANS)^29^, micromagnetic SANS^30^, and a library of physics-based models integrating Monte Carlo simulations and Molecular Dynamics simulations in the SasView^31^ software. Given that a simulation model for the INFER instrument is still in development, none of these existing simulation models could be used at this point.

In addition, one has to consider trade-offs between physics-based and data-driven simulation models. The physics-based models need to have foreknowledge of the hierarchical geometry, materials, and their interactions with neutron beams. The predicted intensities are accurate under the assumptions of the physics model and of the exact knowledge of the experimental setup and variability (noise) sources. In practice, sources of variability are not known, for instance, detector granularity, variations arising from non-parallel wave direction, blurring, instrument design, and others are not known a priori.

The data-driven simulations are limited to the knowledge gained from specific measured datasets and therefore may not extrapolate very well to samples that have not been presented to the data-driven model. Data-driven simulations can learn the variability in the data that is missing in physics-based simulations. Simulating intensities based on data-driven models is typically less computationally expensive than simulating intensities based on physics-based models. The summary of these trade-offs (including an augmentation approach) is provided in Table 1. In this work, we pursue the data-driven modeling approach.

**Table 1:** Summary of trade-offs for a variety of approaches to expanding training datasets.

Approach	Workflow generating training data	Pros	Cons
Manual image segmentation	Collect measurements $\rightarrow$ annotate ROIs	Authentic measurements + experts’ knowledge	High cost of significant experts’ time + labor efforts
Physics-based image simulations	Design physics models $\rightarrow$ prepare geometry & material of sample & scene$\rightarrow$ generate intensity	Low cost of annotation labor	Limited by known physics, approximations, & experimental validation
Augmentations of existing data	Leverage known invariance of image acquisition to generate augmentations	Low cost of augmentations	Very limited dataset expansions due to required a priori knowledge
Data-driven image simulations	Collect reference measurements $\rightarrow$ design estimation and generation models per class $\rightarrow$ prepare geometry of sample & scene $\rightarrow$ generate intensity	Small cost per sample simulation in comparison to real imaging + Dataset expansion space is large in comparison to augmentations	Limited by existing class models and instrument settings, parameter estimation accuracy, & validation of generated intensity

Four approaches for generating synthetic/simulated data are compared based on their data generating workflows and attributes (pros and cons).

### AI model training

Simulated or synthetic data can be used to overcome challenges, such as labeling cost and accuracy^32^, generating large volumes of data, managing privacy and security concerns while allowing usability and transparency of data (e.g., Federal Census data^33^), addressing unbalanced datasets (e.g., Synthetic Minority Oversampling Technique^34^), and generating data that would be unsafe to collect experimentally (e.g., self-driving car accident simulations for training accident-avoidance^35^). A recent press release from Gartner predicts that by 2024, 60 % of the data used for the development of AI and analytics projects will be synthetically generated^36^. By using synthetic data, our work addresses challenges, such as labeling cost, insufficient training data, and class imbalance.

#### Relationship to segmenting hyperspectral images

2D intensity images along the autocorrelation dimension in INFER data is similar in structure to hyperspectral images. This implies that the INFER data can be segmented using segmentation methods developed for hyperspectral images. AI models for hyperspectral image segmentation include 2D or 3D Convolutional Neural Networks (CNNs), Recurrent 2D or 3D CNNs as recommended by Yang et al^37^. Hyperspectral segmentation approaches have been categorized into Object/Superpixel segmentation, Decision fusion, and Feature fusion^38^. Feature fusion methods have generally been shown to be superior but require larger training sample sizes. We leverage the hyperspectral nature of INFER data by using a 2D CNN feature extractor with the Deeplab50^39^ CNN model architecture.

#### Validation of trained AI models

In a 2018 special issue on synthetic data^40^, the editors observed that “There is no free lunch and using synthetic data trades off the manual data acquisition and labeling costs for other generation challenges and a ‘sim2real’ domain gap ”.

The research in the field has been advancing to close the ‘sim2real’ domain gap with better simulation models and augmentation techniques. Domain randomization^41^ is an approach for dealing with this ‘sim2real’ gap via randomization of the properties of each image. This may include greatly varying scene lighting, image quality, object shape and surface properties, as well as the content in the background of the image. In some cases of randomization, performance has been shown to be even better than real data (BDD100K^42^ using structured domain randomization^43^).

A previous study of image-based vehicle detection^44^ explored the viability of training AI models on synthetic images. In this case, synthetic images were created using a physics-based model (i.e., a first-principles ray-tracing model and materials properties defined in terms of their Bidirectional Reflectance Distribution Functions) and used for training the AlexNet CNN model that was pretrained using the ImageNet dataset^45^. In our work, we evaluate similar combinations of train-validation datasets to the reported 2 × 2 combinations, train on {real data, simulated data} × test on {real data, simulated data} by the authors of the vehicle detection system^44^.

In general, many trained AI models suffer from a domain shift problem, which occurs when the validation distribution is different from the training distribution leading to model accuracy decrease^46^. Training on synthetic data and testing on real data (and vice versa) can be viewed as an example of a possible domain shift problem, which is evaluated in our work.

## Experimental results

Our experimental results are divided into (1) validation of data-driven simulations, (2) accuracy evaluations of AI segmentation models, and (3) applicability of data-driven simulations for training AI segmentation models.

To validate statistical data-driven simulations, we first generate a synthetic intensity image using a reference measured mask and estimated parameters. We then perform estimation from this synthetic image. The difference in the parameters estimated from the original versus the simulated images indicates the quality of repeatable data-driven simulations.

To evaluate AI models, we analyze 96 {model, dataset} combinations. We train AI models using measured or synthetic intensity images as inputs and corresponding segmentation masks as outputs. We refer to the segmentation masks as the ground truth (GT). We focus on model accuracy, speed of model training convergence, and model stability. These evaluations aim at choosing optimal input sets and hyperparameters. We also look at whether the models trained on synthetic data are generalizable to measured data and vice-versa.

### Data-driven simulations

Data-driven simulations are evaluated in terms of (a) sufficient dimensionality of the 1D statistical model for modeling 2D images and (b) accuracy of estimated statistical model parameters.

#### Sufficiency of one-dimensional statistical model

**Figure 2:**
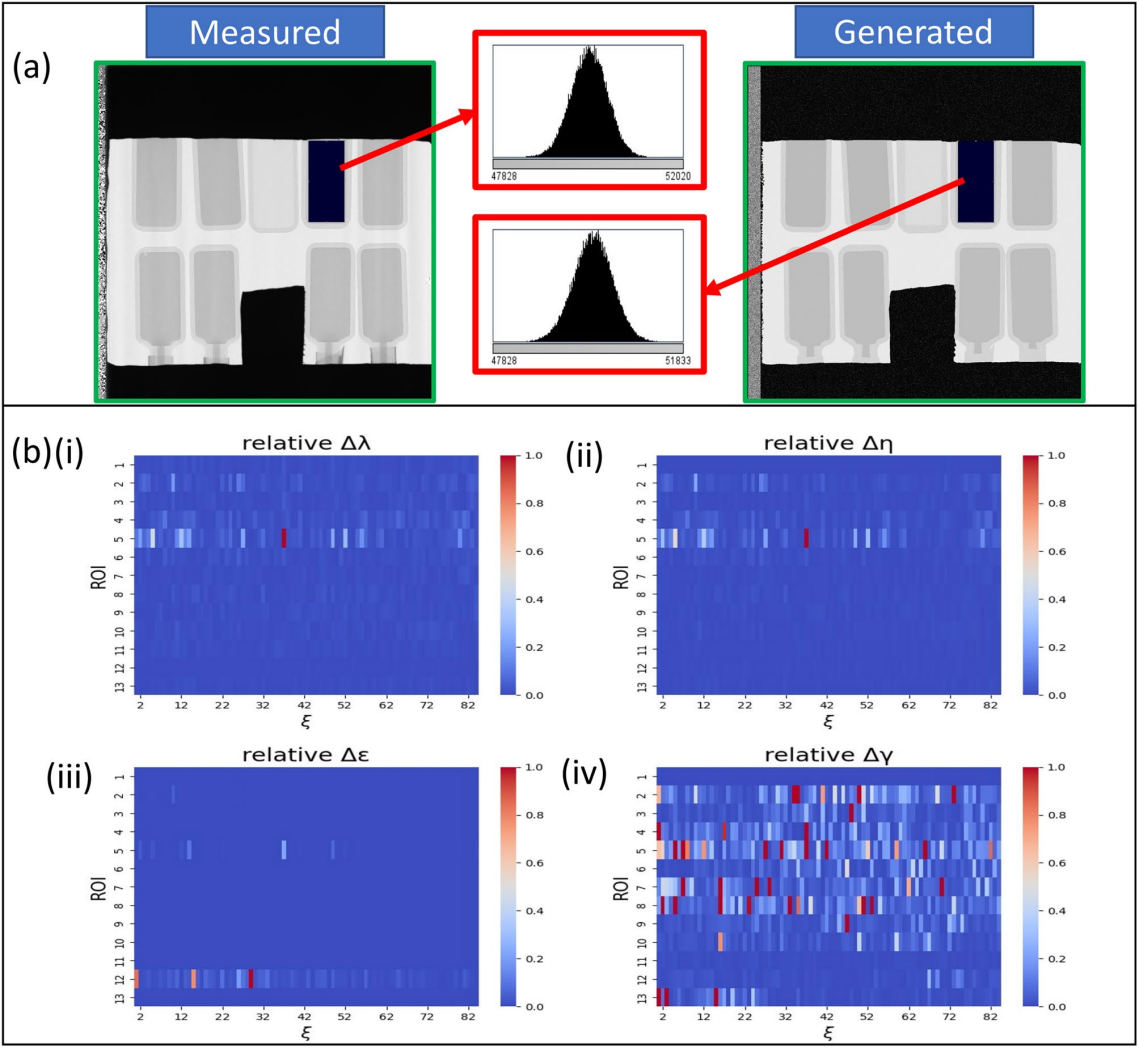
Validation: Data-driven simulations (**a**) Illustration of PDF distributions for the ROI with the label = 4 (the ROI labels are associated with materials in Supplementary Table S2), H1 imaging mode, and autocorrelation = ${41.6416}\,\hbox {nm}$ acquired by the INFER instrument. The mean and standard deviation values for the measured and generated histograms are (49944.6, 407.6) and (49929.7, 419.8) respectively ; (**b**) The four plots show a relative error of statistical model parameters (i) $\lambda$ ,(ii) $\eta$ , (iii) $\epsilon$, and (iv) $\gamma$ as a function of autocorrelation length (horizontal axis) and ROI index (vertical axis). The majority of delta values are close to zero. The gamma parameter shows higher variability on simulated data.

Given the end-goal of simulating 2D images, we explored the 1D versus 2D statistical distributions of intensity values in INFER datasets. We verified that 1D cross-sections of the material-specific ROIs had similar probability density functions (PDFs) of intensity values regardless of each 1D cross-section’s orientation. Due to the very small 2D spatial variation in INFER images for samples such as the one used in this work, a 1D PDF is sufficient for representing intensity statistics that characterize a material ROI. Therefore, neutron INFER data can be simulated by measuring a 1D PDF per 2D ROI. Figure 2a shows comparison of distributions for a selected ROI between measured and generated images. See Supplementary Section 1 for more details.

#### Validation of parameter estimation and image generation during data-driven simulations

To evaluate the accuracy of the data-driven simulations, we computed the delta values according to Eq. (6). The relative error values [see Eq. (7)] are shown in Fig. 2b as a function of the autocorrelation length ($\xi$) shown on the horizontal axis and the region of interest (ROIs) shown on the vertical axis. The calculation method is described in Section “Data-driven image simulations”. We can see clearly that except for a few outliers in the ROI corresponding to material class/label, parameter values of $\lambda$, $\eta$, and $\epsilon$ are very close to the values estimated from the original measured images. The value of $\gamma$ appears to be quite different for all images. However, this can be attributed to the fact that the values of $\gamma$, relating to the horizontal translation of the distribution, are very small (close to 0) in the original estimates and, hence, the normalization by small values causes large fluctuations of the relative metric.

### Evaluation of AI-based image segmentation

We used three evaluation metrics for AI-based image segmentation including accuracy, convergence, and stability as defined in Section “AI model-based image segmentation”.

#### AI model accuracy evaluations

**Figure 3:**
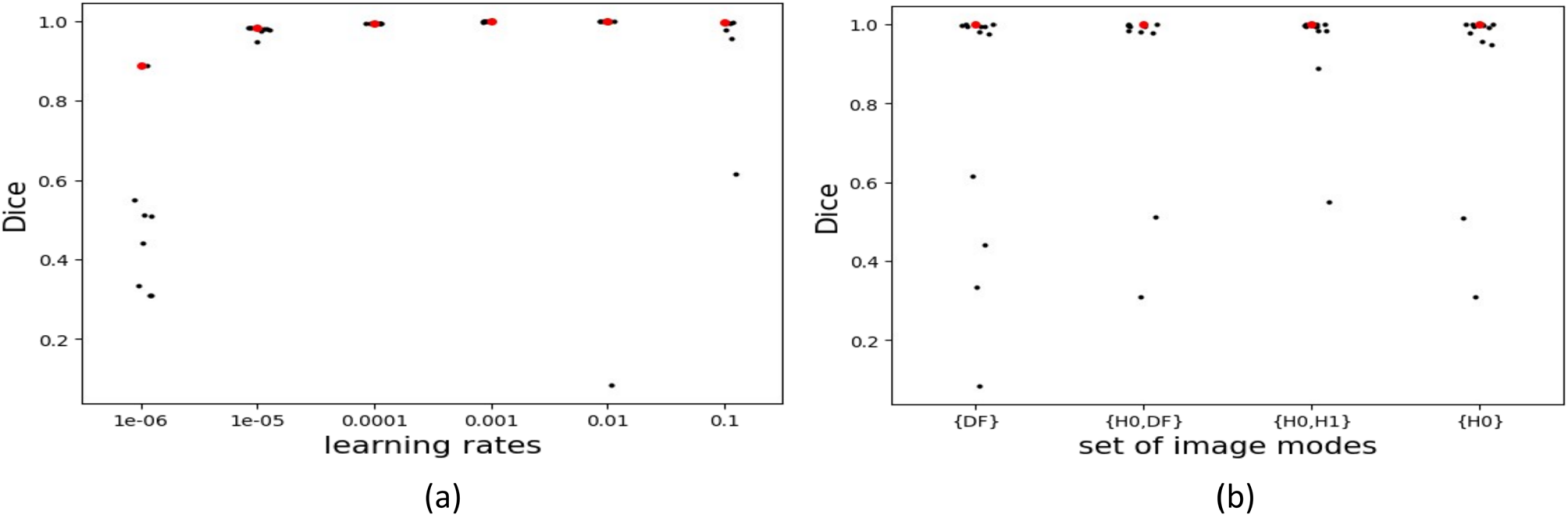
Model accuracy comparison (**a**) : Impact of learning rates (lr) on the AI segmentation model accuracy across all sets of imaging modes and pretraining states. AI segmentation models with maximum Dice score are plotted. They are segregated by set of imaging modes. Each point represents a model with a specific pretrained state and learning rate. The model with optimal Dice coefficient is highlighted in red. According to the Mann–Whitney test (See “AI model-based image segmentation” (**b**)), the learning rate pairs {0.1,0.00001} and {0.1, 0.0001} do not exhibit significant differences, while all other pairs are significantly different to each other.

We evaluated AI model accuracy as a function of a set of imaging mode sets, learning rate, epoch index, and model pretraining. Figure 3 shows that the most accurate models have a learning rate of 0.01. The most accurate models are close to each other with Dice scores close to 0.999. Note that the Dice coefficient is more reliable evaluation metric than cross-entropy (CE) due to it being calculated on different sets of imaging modes (different input data dimensionality) as well as being interpretable metrics for measuring segmentation quality. The results of the highest Dice coefficient among evaluations of 48 hyperparameter combinations over 100 epochs of training are shown in Fig. 3. It shows the Dice index of the optimal model as a function of imaging mode sets.

Given the results, we made three observations. First, Fig. 3a shows that learning rates have a significant impact on the model accuracy obtained during any training. Note that the most accurate model is selected also across all epochs. Learning rate of 0.01 was the most optimal during our runs, very closely followed by 0.001. Second, Fig. 3b shows that choosing a different set of imaging modes does not significantly change the average across different learning rates and pretraining states. This implies that any combination of imaging modes in our experimental design is appropriate for segmentation. Third, in terms of an epoch index, the epoch for the highest accuracy model usually occurs in the 90-100 epoch range consistently. Between the set of imaging modes chosen, the values of the Dice coefficients appear to be very close to each other with {H0,H1} and {DF} since they are mathematically related $DF = -ln(H1/H0)$.

Finally, the accuracy values between pretrained models on the COCO dataset and randomly initialized DeepLab50 model coefficients are statistically not significant (see Supplementary Fig. S6c) due to very limited commonalities between COCO and INFER datasets.

#### AI model convergence speed evaluations

**Figure 4:**
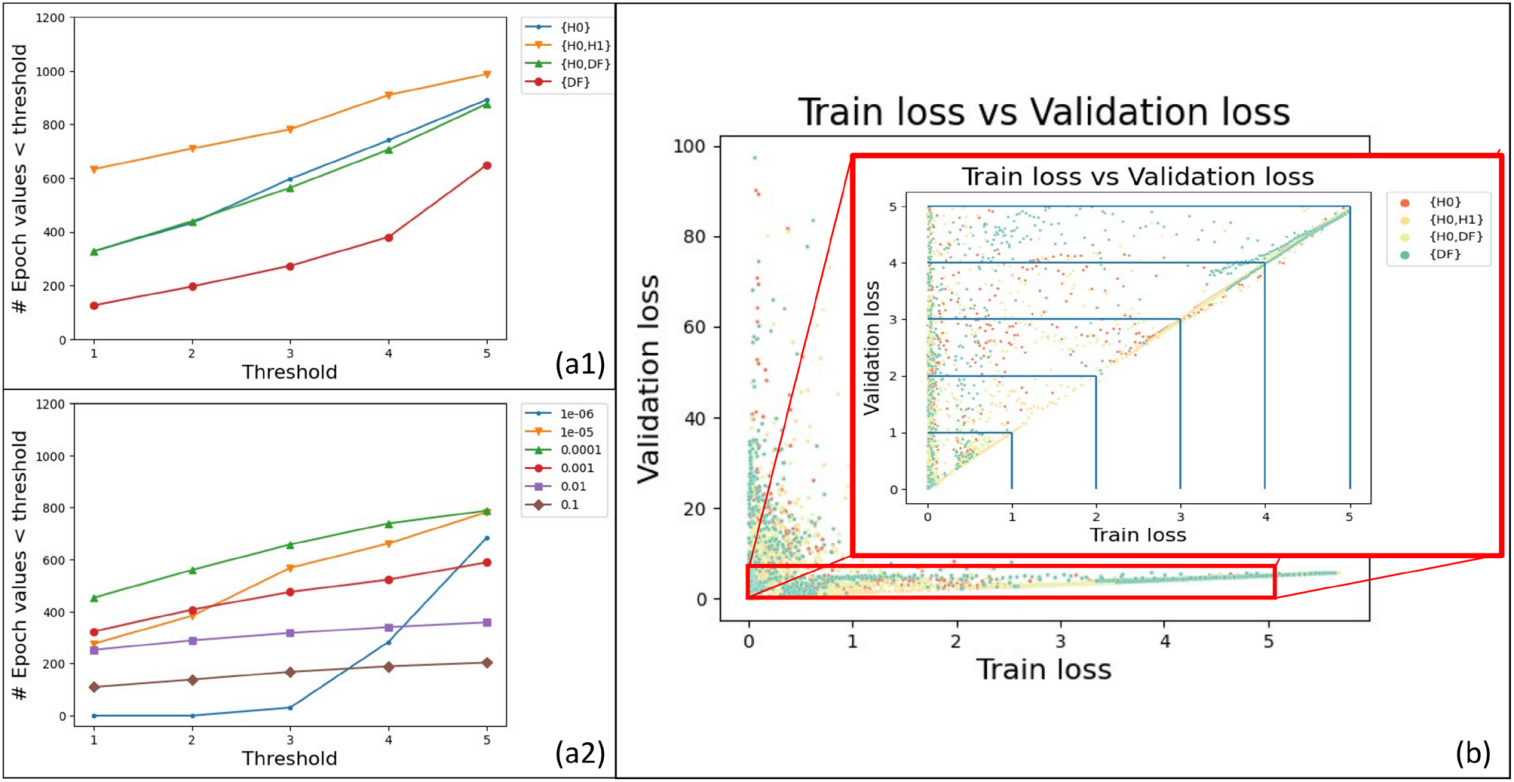
Model convergence speed metrics (**a**) Convergence speed evaluation using the number of epochs with lower CE than a threshold as a function of the set of imaging modes (1) or a set of learning rates (2). The threshold refers to the value for CE loss (both Train and Validation) according to Eq. (9) (**b**) Train vs Validation loss (CE). Inset: Magnified view of CE Validation loss and CE Train loss in the range of [0,5]. Blue lines indicate thresholds below which number of models were counted.

We explored the AI Model convergence speed as a function of the set of imaging modes combinations, learning rate values, and model initialization {pretrained, random}. The first two functions are documented in Figure 4.

First, in our experiments, the set of imaging modes combination {H0, H1} appears to have the most epochs under the CE loss value of five for both train and validation subsets. This combination shows consistently higher convergence rates regardless of the choice of a CE loss threshold. This indicates that models using {H0, H1} imaging modes as inputs converge the fastest regardless of pretraining or learning rates. Second, regarding the learning rates, model convergence speed increased while learning rates are changing from $10^{-6}$ up to $10^{-4}$ before decreasing for increasing learning rates larger than $10^{-4}$. Finally, pretraining the AI model DeepLab50 using the COCO dataset had no discernible effect on the AI model convergence speed (see Supplementary Fig. S5a).

#### AI model stability evaluations

Model stability was calculated with respect to the set of imaging modes, learning rates, and pretraining state according to Section “AI model-based image segmentation”. For each calculation, data were also split along one of the three parameters (sets of imaging modes, learning rates, pretrained models) to obtain insights.

First, when comparing the set of imaging modes, the residuals follow similar trends to model accuracy evaluations. As can be seen from Supplementary Fig. S6b, no significant difference is observed between the different sets of imaging modes based on the Mann–Whitney statistical test. Second, AI model stability is higher for lower learning rates as expected. Residuals are very small for low learning rates and go up as much as 14 orders of magnitudes for high learning rates. This is primarily due to a few outliers in the CE loss values. For example, a single outlier value where CE = $10^{16}$ causes the entire shift in average residual. While only a handful of such outliers exists in these high learning rate models, we can see in Supplementary Fig.  S6a the trend observed as a function of learning rate remains consistent. Finally, pretraining an AI model on the COCO dataset did not significantly change the model stability metric as can be seen in Fig. S6c.

### Applicability of data-driven simulations for training AI segmentation models

The overarching goal of this work is to use data-driven simulations for training an AI model that will accurately infer image segmentation of measured images. To fully evaluate the performance of a trained AI model, we conducted experiments with four combinations of training and validation (evaluations) on data-driven and measured image sets. Table 2 shows the experimental results of the four conducted experiments.

**Figure 5:**
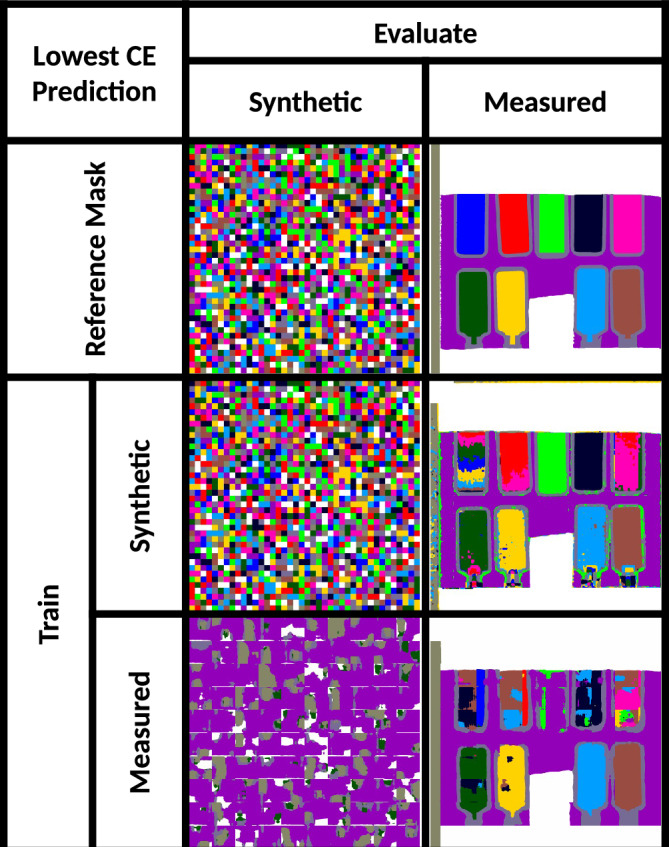
Train-evaluation pairs for optimal models. Segmentation masks obtained with the most accurate AI model for each train/evaluate pair. The row with the Reference Mask shows GT masks for synthetic and measured images. The four images below the masks at the intersection of Train rows and Evaluate columns correspond to the four combinations of training and evaluation datasets and should be compared with the reference masks.

**Table 2:** Segmentation quality metrics.

		Evaluate	
		Synthetic	Measured
Dice			
Train	Synthetic	0.9995	0.8572
	Measured	0.1842	0.8365
IoU score			
Train	Synthetic	0.9989	0.7811
	Measured	0.1040	0.7739

Comparison of Dice and IoU scores for the best (highest Dice score) model when trained on data-driven or measured and tested on data-driven or measured datasets. Best models selected using the highest Dice score.

In the four experiments, we trained 48 AI segmentation models on simulated data and 48 models on measured data using the same AI model architecture. We used all measured image tiles for training since the measured data were limited to $1924 \times 1924$ pixels. For the comparisons, both the generated data-driven and measured datasets for the same values of autocorrelation and imaging mode sets were assembled.

While the accuracy results for the overarching goal are satisfactory (train on data-driven and evaluate on measured), the accuracy values for “train on measured and evaluate on data-driven” are surprisingly low. We observe qualitatively that for the CNN model trained on measured data and evaluated on synthetic checkerboard data, the model predicts large connected regions as learned from the measured training data. We hypothesize that the CNN model has learned from measured data that the single-label regions are much larger than simulated checkers. To confirm the hypothesis, a micro-average Dice score was calculated for predicted segmentation masks per AI model—see the Dice Eq. (8) in the “Methods” section.

The resulting micro-averaged Dice scores are the highest for the three background labels, and to a lesser extent for the quartz sample holder. One can also observe in Fig. 5 that the background labels are over-represented in predicted segmentation masks when an AI model trained on measured data is evaluated on simulated data. These biases may be occurring because of (a) an imbalance in material classes when training on measured data and (b) the 12 × smaller size of measured dataset than the size of the simulated dataset.

## Discussion

We briefly discuss the lessons learned from the experimental results and the applicability limits of the presented approach. A choice of a set of imaging modes did not appear to impact the accuracy of AI-based segmentation in a statistically significant way. We anticipated that {H0, H1}, {DF}, or {H0, DF} input sets would improve segmentation accuracy since the inputs contain more information than the {H0} mode. It turned out that the current sample contained microstructures characterized by the attenuation model H0. In general, we do not know whether a sample has microstructures distinguishable only using the first imaging mode H1 or not. Therefore, the optimal input set would be expected to be{DF} since it combines information from H0 and H1 and has a minimal cardinality of the sets. Instead, learning rates were found to have the highest impact on model convergence speed and accuracy with optimal values being 0.001 and 0.0001 for the experimental setup.

Initializing AI models with a COCO pretrained model did not improve the accuracy of AI-based segmentation in a statistically significant way. This result implies that the segmentation class characteristics in INFER images do not overlap with the class characteristics in COCO images (person, bicycle, tree, road, etc.). A similar result was obtained for pretrained models previously^28^. The optimal configuration was found to be a random initialization of an AI model.

An interesting observation from Fig. 5 is that samples with index 4 and 11 appear on the quartz holder border; this can be explained from the fact that index 4 is empty quartz holder control and is, therefore, very similar to the quartz holder. This shows that many instances of mislabeling by the model are explainable. Another observation is in the prediction of index 2 where we see layers with different labels. This was found to be due to creaming in the sample with large size ($\hbox {d}={1000}\,\hbox {nm}$) of polystyrene beads. Creaming is the migration of dispersed phase of an emulsion under the effect of buoyancy which forms a gradient of concentrations. The AI model segments the region into discrete labels appearing as layers. Although the AI model appears to be mislabeling a single GT region, it is actually capturing a phenomenon that was not planned before the experiment. It shows that the GT labels for ROI 2 were not correct. Unfortunately, the model cannot discriminate between labels assigned due to size variation versus concentration gradient, but it can highlight the creaming phenomenon during segmentation quality inspection. Further analysis on creaming can be found in Supplementary Section 7.

**Figure 6:**
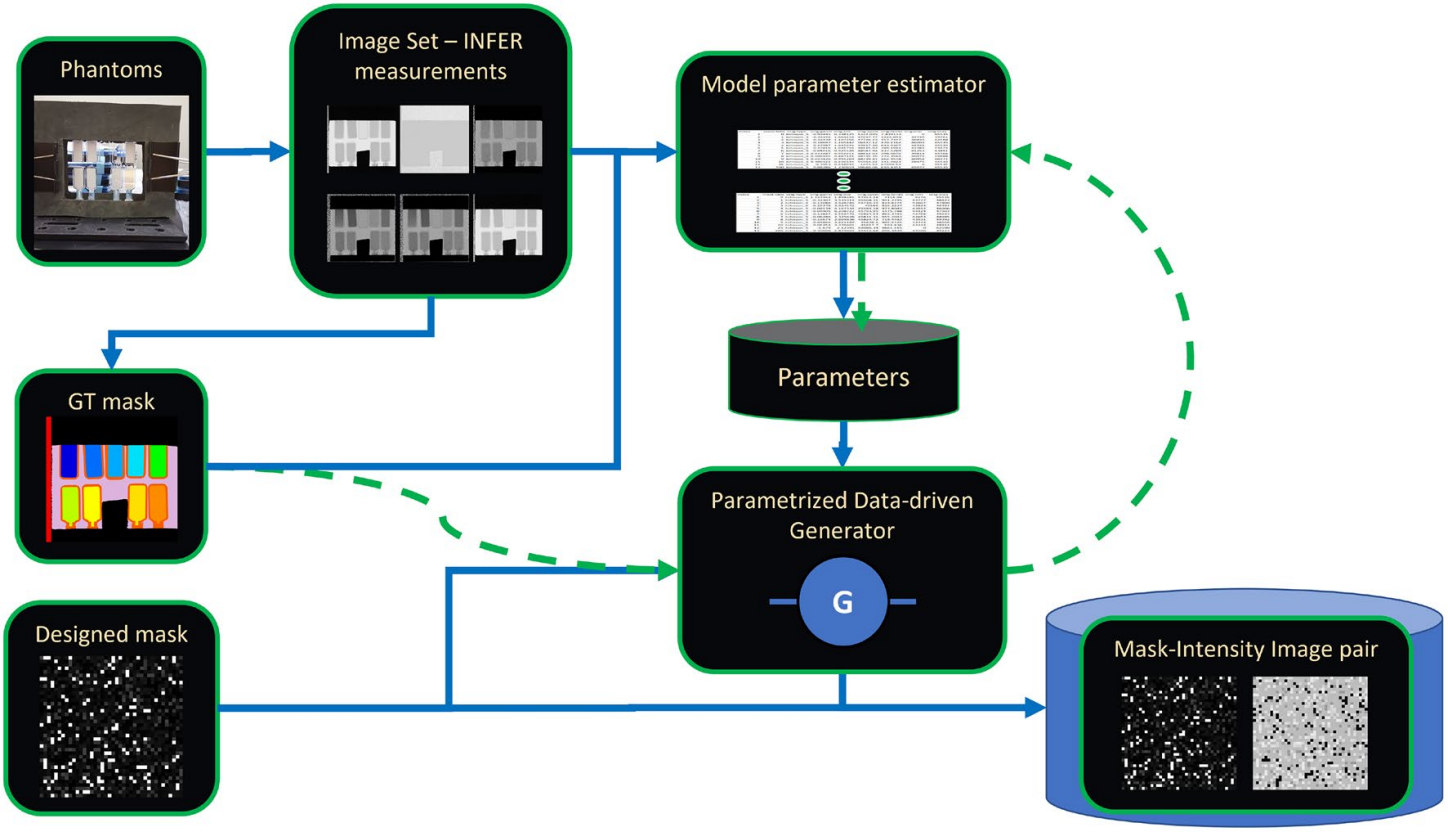
Data-driven simulation workflow. Dashed green lines indicate validation. Blue lines indicate the process starting from phantom measurement to generation of mask-intensity image pairs.

The computational workflow shown in Fig. 6 is fully automated except for creating a segmentation mask for measured images. We have validated that the Johnson family of 1D PDFs successfully captured statistics of 2D INFER images. However, we observed that the Johnson family is not suitable for modeling uncorrected images with Moiré fringes acquired using the 2-grating far field interferometer nor for modeling the periodic patterns in extracted differential phase contrast ($\phi$) images (angle of neutrons passing through the grating). If a sample/scene segmentation task were needed to leverage the uncorrected or $\phi$ images, then the underlying data-driven model would have to change.

The numerical results indicate that using data-driven simulations for training an AI model is a viable option for accurately inferring image segmentation of measured images. An interesting outcome of this work is that checkerboard patterns as scene simulations yielded a more accurate trained AI models than augmented scene simulations as well as measured data. Due to the lack of measured data, we have only been able to validate the viability with one experimental setup, which limits the evaluation robustness. In the future, segmentation accuracy validations with other experimental setups will improve our quantitative understanding of the approach viability.

## Conclusions and future work

We designed data-driven simulations for neutron interferometry and evaluated accuracy of AI-based segmentation models trained on data-driven simulations and measured datasets. We concluded that data-driven simulations of neutron INFER imaging data improve accuracy of a trained AI model for image segmentation tasks and can be employed when there is a scarcity of measured data. Furthermore, simulations and pretrained AI segmentation models can also assist in steerable experiments. In our experiments, training AI models using data-driven simulations outperformed training AI models using measured data by mitigating spatial biases inherently encoded in limited measurements.

Our work poses several interesting future directions in terms of mixing physics-based and data-driven approaches and physics-informed neural networks, as well as exploring other data-driven models. Additional exploration of data-driven simulations for the intra- or inter-sample image comparisons at the same or different neutron beam locations will help in better understanding the limitations of data-driven simulations. The discovery of a phenomenon like creaming, that the model is not trained on, opens up questions about sensitivity of the data and the AI models to variability in spatial distribution of correlograms. Finally, as illustrated with our clustering results in the Supplementary Section 6, future work will explore measurement baselines that can identify any experimental deviations from theoretically expected characteristics of reference materials.

## Methods

In this work, we designed a methodology based on data-driven simulations and image segmentation methods. The data-driven simulations are beyond typical image augmentations used for expanding datasets during AI model training. It is assumed that at least one measurement with known segmentation into semantically meaningful classes is available for training an AI segmentation model.

The data-driven simulation method is based on a statistical model for intensity distributions. The use of a statistical model in our work consists of four steps: (a) model parameter estimation from measured images and their corresponding material-specific image masks, (b) generate simulated image masks representing geometrically perturbed distributions of materials, (c) generate intensities over image masks based on parameterized statistical models, and (d) evaluate the simulation error by comparing estimated statistical model parameters from simulated images and the initially estimated model parameters from measured images.

The image segmentation method is based on supervised AI models designed for image segmentation tasks. In addition to standard training and validation steps in AI-based modeling, our segmentation workflow includes (a) optimization over sets of INFER imaging modes and (b) evaluations of segmentation accuracy, training convergence, and model stability for AI models trained on simulated or measured images.

### Materials and data

The materials used in our experiments are well-characterized polystyrene (PS) suspensions in Deuterium Oxide (${\hbox {D}_{2}\hbox {O}}$). The diameter size of PS beads is varied while keeping the scale/volume fraction constant. Each of the nine rectangular quartz cuvettes has a pathlength of 2 mm. Out of nine samples, two control samples: one with pure ${\hbox {D}_{2}\hbox {O}}$ solution and another with an empty quartz cuvette, are also present. Supplementary Fig. S2 and Table S2 describe the samples in more detail (Supplementary Section 2).

In order to develop and verify our data-driven simulation, we imaged well-characterized materials (i.e., measurement phantoms) using the CG-1D^47^ neutron beamline at the Oak Ridge National Laboratory (ORNL) High Flux Isotope Reactor (HFIR). Data are obtained as 16-bits per pixel grayscale images stored in the Tiff file format. The acquisition protocol and the instrument setup are described by Kim et al.^48^. Images are obtained for two imaging modes and 84 autocorrelation lengths based on the method described by Wen et al.^22^. We denote *H*, $\Xi$, *X*, *Y* as the four dimensions of the acquired data, where imaging mode (*H*), autocorrelation length ($\Xi$) , and spatial coordinates (*X* and *Y*) refer to each dimension. The segmentation problem can be described mathematically as a mapping from the input dataset (*H*, $\Xi$, *X*, *Y*) to (*X*, *Y*) where values in the *H* and $\Xi$ dimensions are replaced with a semantic class label according to value similarities and spatial proximity (see Eq. 1 below), where *v* refers to grayscale values and *c* to semantic classes.

1 $$\begin{aligned} (H,\Xi , X, Y)_v \rightarrow (X, Y)_c \end{aligned}$$

When validating our data-driven simulations, we denote the measured image $I_{h,\xi }^{meas}$ and the simulated image $I_{h,\xi }^{sim}$ , where *I* is the image intensity, $h \in H$ is the imaging mode along the *H* dimension, and $\xi \in \Xi$ is the autocorrelation length along the $\Xi$ dimension. The superscript *meas* and *sim* indicate measured and simulated images, respectively.

To correct raw image data for geometric and optical distortions, our reconstruction approach is based on Kim et al.^48^ and we adopt the nomenclature therein. Imaging mode H0 is intensity, and H0 normalized to the open beam signal it becomes transmission. H1 is the Visibility and $-ln(H1/H0)$ is Dark-Field (DF). One can view the input dataset as a hyperspectral cube with dimensions ($\Xi \cup H$, X, Y) by concatenating values along the autocorrelation and imaging mode dimensions. These 3D data become the input to our AI model for a semantic segmentation task.

### Data-driven image simulations

First, we present the statistical model for estimating and generating intensities and the data-driven simulation framework. Next, we overview the image mask creation for which intensities are simulated. Finally, we validate the simulated images against the original images using statistical model parameters.

#### Statistical model for estimating and generating intensities

The statistical model represents image intensities with the Johnson family^49^ of probability density functions (PDFs) defined in Eq. (2) below. In this equation, variable *x* represents $I_{h,\xi }^{sim}$ and the four parameters are estimated from the original intensities, $I_{h,\xi }^{meas}$. The PDF model is based on a set of transformations from a normal probability distribution. The four parameters can be viewed as offset $\gamma$, scale $\eta$, shift $\epsilon$, and spread $\lambda$. The Johnson family of PDFs can be interpreted as a set of basis functions $\tau$ defined in Aupplementary Table S1.

2 $$\begin{aligned} x = \gamma + \eta * \tau \left( \dfrac{z-\epsilon }{\lambda } \right) ; \quad z \sim N(0,1) \end{aligned}$$

#### A data-driven simulation workflow

Based on the statistical model, Fig. 6 describes the data-driven image simulation workflow. The simulation workflow starts with creating a segmentation mask for measured phantom samples (GT Mask in Fig. 6) by using ImageJ^50^. The mask creation is described in Supplementary Section 2. In our work, the mask of material-specific ROIs was created manually from the H0 imaging mode (transmission) because the H0 images had the highest contrast. The mask delineates ROIs for three background types, two control, and eight material samples. The quality of the mask was inspected visually by multiple experts.

In Fig. 6’s “Model parameter estimator” the statistical model parameters are estimated for each ROI in each image and stored in a file. For our measured dataset, there are 252 sets of four parameters estimated for 84 autocorrelation lengths and two imaging modes and a derived ratio of modes (Dark-Field or $\{DF\}$) per ROI.

In “Parametrized data-driven generator” shown in Fig. 6, an image mask is loaded and populated with intensity values per ROI by using the data-driven model and the label assigned to each ROI. Each mask label is associated with a unique triplet (material type, H0 and H1 imaging modes, auto-correlation value). The mask label is used as an index to retrieve parameters of a statistical model. The intensity values are trimmed to minimum and maximum values in the measured ROIs.

#### Design of simulated scenes (image masks)

Geometric configurations of imaged physical samples are denoted as imaged scenes. They can be represented by image masks or 2D image projections consisting of ROIs associated with unique labels that correspond to a unique material type. The designed image masks or “Designed Masks” as shown in 6 can be created using three approaches: (1) permutation of materials and perturbation of ROIs in measured images, (2) designing anticipated geometric configurations of physical samples, and (3) imposing a class balancing constraint (an equal class label representation) on simulated scenes to avoid class prediction bias in trained AI models.

Image mask design by material permutations and spatial location perturbations: (1) material permutations are achieved by randomly assigning labels to existing ROIs. These permutations reduce the chance of learning any association between ROI and material type. (2) location perturbations are introduced by randomly translating ROIs in any direction for up to a given maximum value. These perturbations encode translational invariance in an AI model. Supplementary Fig. S3a shows an example of a generated mask using permutations and perturbations. This scene design approach assumes that future experiments will analyze similar geometric configurations of imaged samples to the one already measured.

Image mask design based on anticipated geometric configurations of physical samples: A scene is designed by placing a set of primitive shapes representing ROIs according to composition rules that mimic the anticipated physical sample composition. Supplementary Fig. S3b shows two examples of image masks simulating a sample as a container with a grid of beads made from unique materials (left) and with randomly fused beads of varying diameters and material types (right). This scene design approach assumes that future experiments will analyze samples with known materials and anticipated spatial deviations from the measured sample composition.

Image mask design with a class balancing constraint: a scene is designed by forming a checkerboard pattern of ROIs in a mask image and class label assignment to ROIs so that mask sub-regions contain equal numbers of pixels from all classes. The size of checkers is selected based on the following objectives: (a) a mask sub-region with its corresponding intensity subregion must fit into the random access memory (RAM) of the graphics processing unit (GPU) used for AI model training, (b) all class labels must be uniformly represented in a mask sub-region, and (c) predicted mask sub-regions must be visually simple to compare with simulated GT mask (i.e., pattern granularity is sufficiently large).

In order to meet the constraints, the class labels are selected uniformly. The size of checker *D* and the batch size $S_{batch}$ must be chosen to satisfy these constraints. Equation (3) provides a relationship for each tile to contain each label at least once (on average). The equation variables refer to image sub-region (tile) size ($S_{Tile}$), number of classes (C), and checker width (D).

3 $$\begin{aligned} D \le \left\lfloor \sqrt{\dfrac{S_{Tile}}{C}} \right\rfloor \end{aligned}$$

For our work, $C=13$ and $S_{Tile}=192$, which gives:

4 $$\begin{aligned} D \le \left\lfloor \sqrt{\dfrac{192 x 192}{13}} \right\rfloor = \lfloor 53.2512\rfloor \quad \quad \quad i.e.,\quad D \le 53 \end{aligned}$$

For visual verification, the choice of *D* is subject to $D\ge 10$ (selected subjectively). Our checker width is 50, which fulfills these criteria. On the scale of the entire checkerboard, the number of checkers for each label were measured to be in the range 78–120, averaging exactly 100.

To choose ($S_{batch}$), the Eq. (5) below provides a relationship between the GPU RAM size ($S_{GPU\_RAM}$), batch size ($S_{batch}$), bits per pixel (BPP) for mask ($BPP_{mask}$) and intensity ($BPP_{intensity}$), and the number of imaging modes ($N_{H}$). As the model architecture size and the computational code occupy some RAM, our batch size varied to maximize the GPU RAM usage $S_{GPU\_RAM}$ (in our case, $S_{GPU\_RAM}=80~Gb$). This requirement is modified slightly when considering low amounts of data, when dividing data into appropriate sized parts takes precedence—due to which the value is 20 for training on measured data to allow 80–20 division when total tiles = 100.

5 $$\begin{aligned} S_{GPU\_RAM} \propto S_{batch} * S_{Tile} (BPP_{mask}+N_{H}*BPP_{intensity}) \end{aligned}$$

Supplementary Fig. S4 shows two examples of image mask designs with a material class balancing constraint. Both masks in Figure 3 satisfy the constraint and differ by random locations and neighboring relationships of material class labels. This scene design approach assumes that the segmentation task can be simplified to a clustering task with intensity dependencies constrained to a small spatial neighborhood.

#### Validation of data-driven simulation

The validation of data-driven simulations is performed by comparing estimated parameters from simulated and measured images using the same image mask that was created for the measured images. The evaluations compare Johnson’s family type mismatch and the deltas for all parameter values. The absolute and relative deltas are the difference between the parameters obtained from intensity values of $I_{h,\xi }^{sim}$ and $I_{h,\xi }^{meas}$. They are defined in Eqs. (6) and (7) below, where, *Var* can be any of the parameters: $\gamma$ (gamma), $\eta$ (eta), $\epsilon$ (epsilon), $\lambda$ (lambda). The $\Delta Var$ values reflect the quality of simulated intensities according to the estimated PDFs from measured data.

6 $$\begin{aligned} \Delta Var&= Var \left( I_{h,\xi }^{sim} \right) - Var \left( I_{h,\xi }^{meas} \right) \end{aligned}$$

7 $$\begin{aligned} relative \left( \Delta Var \right)&= \left| \dfrac{\Delta Var}{Var \left( I_{h,\xi }^{meas} \right) }\right| \end{aligned}$$

### AI model-based image segmentation

First, we describe a feature extractor to accommodate variable input dimensions and introduce AI model hyperparameters to be optimized during AI model training. Next, we overview the space of evaluation configurations and present evaluation metrics.

#### Feature extractor for variable input dimensions

According to the neutron physics^6^, the Dark-Field values $DF = -ln(H1/H0)$ combine the discriminatory power of the H0 and H1 imaging modes for material characterization. To understand the value of imaging modes for image segmentation tasks, our study also investigated the accuracy of trained AI models as a function of combinations of the H0 and H1 imaging modes. We constructed the set of {H0, H1}, {DF}, {H0, DF}, and {H0} of inputs into AI model training, where {H0} was included as a baseline.

Our investigation of sets of one or two imaging modes introduced a varying number of inputs into AI model training. Furthermore, specific autocorrelation lengths and their total numbers vary across experiments and must be considered when preparing inputs for AI model training. To address this variability of inputs, we designed a feature extractor step to map data from $1 \times 84$ ({1 imaging mode} × {84 autocorrelation lengths} = 84) or $2 \times 84$ ({2 imaging modes} × {84 autocorrelation lengths}) dimensional inputs to a $1 \times 3$ dimensional inputs depending on the set of imaging modes included in {H0}, {H0,H1}, {DF}, and {H0,DF}. We add an extra model parameter, which allows us to change the number of inputs depending on which element of the set is considered.

#### Hyperparameter optimization of AI segmentation models

For the $1 \times 3$ dimensional inputs after feature extraction, we previously^28^ empirically evaluated 6 AI CNN architectures available in the PyTorch^51^ library: DeepLab 50, DeepLab 101, MobileNetV3-Large, LR-ASPP-MobileNetV3-Large, FCN ResNet 50 and FCN ResNet 101. Based on our observations, we selected the DeepLab50 architecture in this work. In addition to exploring 4 combinations of H0 and H1 input modes in a set of {H0, H1}, {DF}, {H0, DF}, and {H0}, we sampled the learning rate and the random or COCO^27^ pretrained initialization of the AI models. The following learning rates were tested for optimization: {$10^{-6}, 10^{-5}, 10^{-4}, 10^{-3}, 10^{-2}, 10^{-1}$} with the Adam Optimizer.

**Figure 7:**
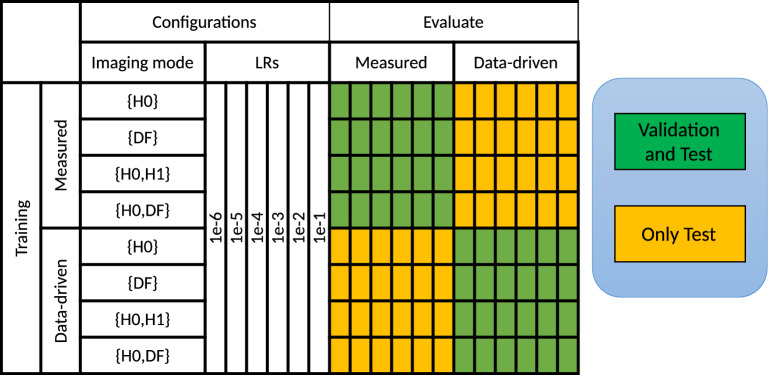
Design of AI model train-test experiments. Summary of experimental configurations with measured and data-driven (synthetic) images. Each square also has a pretrained/not-pretrained (randomly initialized) model on COCO dataset. LRs stands for learning rates.

See Fig. 7 for a summary of experimental configurations tested. A simulated data test set was generated using 15 different checkerboards. Validation set used during training is always of the same type, for example, when training on simulated data, validation set is also simulated data. Inference was run on Test set for all combinations of parameters, thus allowing us to compare the methods.

#### Measured vs simulated evaluations of AI segmentation models

In order to establish the value of data-driven simulations, we explored four combinations of AI models: (1) trained on measured data and evaluated on measured data, (2) trained on data-driven simulation and evaluated on measured data, (3) trained on measured data and evaluated on data-driven simulation data, (4) trained on data-driven simulation and evaluated on data-driven simulation. In cases where the measured training data are severely constrained, the minimum number of tiles per batch must be greater or equal than the number of tiles available for validation. In our case, we choose the batch size to be 20 tiles due to the limited number of measured tiles equal to 100 and their split to 4*20 train and 20 validation tiles.

#### Evaluation metrics of AI segmentation model

We evaluate trained AI segmentation models based on model segmentation accuracy, training convergence, and model stability for the four combinations of AI models trained on simulated or measured images.

*Model accuracy* To find the most accurate models, we use the Dice coefficient^52^ since it measures directly the quality of the predicted segmentation mask. The definition of Dice score is shown in Eq. (8). The most accurate model is recorded over 100 epochs of training.

8 $$\begin{aligned} Dice = \frac{1}{tiles}\sum _{i=1}^{tiles}\frac{2 \times \sum _{j=1}^{labels} TP_{ij} }{ \sum _{j=1}^{labels} (2 \times TP_{ij} + FP_{ij} + FN_{ij}) } \end{aligned}$$

where $TP_{ij}$, $TN_{ij}$, $FP_{ij}$ and $FN_{ij}$ are abbreviations for True Positive, True Negative, False Positive and False Negative respectively and subscripts *i* and *j* indicate tile *i* and label *j*.

*Model training convergence* We calculate a root-mean-squared error (RMSE) metric over training and testing CE errors shown in Eq. (9):

9 $$\begin{aligned} CE loss = \sqrt{CE_{train}^2 + CE_{validation}^2} \text {subject to} \quad CE_{train} \le thresh \quad \& \quad CE_{validation} \le thresh \end{aligned}$$

Minimizing the RMSE metric over all epochs provides an insight into the convergence speed of each model training. Furthermore, we can analyze the number of epochs when a model was contained in magnitude-constrained regions as illustrated in Fig. 4. If many models represented by train and validation CE loss lie within a magnitude-constrained region, then the model was able to reach CE losses below the magnitude threshold at an earlier epoch and, hence, converged faster. Figure 4 shows the CE loss constrained regions by values 1, 2, 3, 4, and 5 (delineated by the blue lines). The color-coded points correspond to the AI models at each epoch contained by these regions for the four sets of imaging modes as inputs.

*Model stability* Due to the complexity of the non-linear functions relating inputs and outputs in a model, optimization may yield a highly accurate model that is very unstable as the parameters change. To measure model stability, we assume that a stable model would have a linear dependency between train and validation CE loss. We calculate the residual/error of the least squares linear fit to CE train vs CE validation losses as a measure of model stability. The higher the residual, the lower the fit quality. In general, a higher stability or lower residual should be preferred.

*Statistical significance of metrics* Model metrics for varying inputs are compared with each other using the Mann–Whitney U test^53^. This Mann–Whitney statistical U-test evaluates the hypothesis that the probability distribution of a randomly drawn observation from one group versus the one from the other group is the same. When comparing values, such as model CE loss, distributions are non-normal and occasional spikes in model training add outliers. We selected the Mann–Whitney U-test because it does not assume normality of CE loss data. It is also robust to outliers as it relies on ranks unlike the t-test.

### Supplementary Information

Supplementary Information.

## Data Availability

The Datasets are available from https://isg.nist.gov/deepzoomweb/data/inferSegmentation.
