# Supplementary material for: Data-driven simulations for training AI-based segmentation of neutron images
Source: Sci Rep. 2024 Mar 19;14:6614. doi: 10.1038/s41598-024-56409-3 (PMC10951284; doi:10.1038/s41598-024-56409-3)
Supplement: Supplementary file 1 — Supplementary Information. [file 41598_2024_56409_MOESM1_ESM.pdf]

Supplementary information for the paper entitled *Data-driven Simulations For Training AI-Based Segmentation of Neutron Images*

Pushkar S. Sathe[1,*], Caitlyn M. Wolf[2], Youngju Kim[3,4], Sarah M. Robinson[3], M. Cyrus Daugherty[3], Ryan P. Murphy[2], Jacob M. LaManna[3], Michael G. Huber[3], David L. Jacobson[3], Paul A. Kienzle[2], Katie M. Weigandt[2], Nikolai N. Klimov[3], Daniel S. Hussey[3], and Peter Bajcsy[1,*,+]

[1]Information Technology Laboratory, NIST, Gaithersburg, Maryland - 20899, USA
[2]NIST Center for Neutron Research, Gaithersburg, Maryland - 20899, USA
[3]Physical Measurement Laboratory, Gaithersburg, Maryland - 20899, USA
[4]Department of Chemistry and Biochemistry, University of Maryland, College Park, Maryland - 20742, USA
[*]These authors contributed equally to this work
[+]peter.bajcsy@nist.gov

ABSTRACT

In this supplementary document, we present additional Figures, Tables, and Discussion about the Data-driven Simulations For Training AI-Based Segmentation of Neutron Images.

1 Johnson family of probability density functions (PDFs)

Table S1 presents four distribution models in the Johnson family of PDFs used in our data-driven simulations. As we can see from Figure S1, the histograms of uniform materials such as the selected ROI (See Figure S2 and Table S2, index 5) appear to have very similar distributions regardless of the directional bias of the region of interest (ROI). Using a large number of pixels makes the histogram distributions smoother and more reliable estimations (Figure S1(e)). The standard deviations also converge to the same value as larger number of pixels are included in the ROI. This confirms that there is no directional bias in the 2D ROIs as expected from the experimental setup.

Johnson's S_B-distribution	$\tau(z) = (1+exp(-z))^{-1}$
Johnson's S_U-distribution	$\tau(z) = sinh(z)$
Johnson's S_L-distribution	$\tau(z) = exp(z)$
Johnson's S_N-distribution	$\tau(z) = z$

Table S1. Members of Johnson's family of probability density functions (PDFs)

2 Sample information and mask generation

The mask is generated manually using ImageJ/Fiji. A projected mean is obtained from the stack of images along the $H \bigcup \Xi$ dimensions as a starting point. Figure S2 shows a visualization of unique materials following a look-up color table after the mask is completed from measured data manually. Index refers to the ROI number presented in the figure. The actual 8-bit label can be different and this can be specified in a mapping csv file that is used in the Estimation and Generation steps. Carboxyl-functionalized polystyrene nanoparticles dispersed in water were purchased from Bangs Laboratories, Inc. with nominal diameters of 50, 65, 101, 145, 198, 500, and 1000 nm. Deuterium oxide (D_2O) was purchased from Cambridge Isotope Laboratories and sodium dodecyl sulfate (SDS) was purchased from Millipore Sigma. A 3.5 mM solution of SDS in D_2O was prepared for solvent exchange of the polystyrene nanoparticle dispersions. The solvent exchange was performed using Millipore Ultra Centrifugal Filter tubes. The final concentration of the polystyere nanoparticle dispersions in heavy water were adjusted to 5 % by volume for all particle sizes. Note that three different backgrounds are labelled (indices 1, 12, 13 that correspond to semantically different materials as documented in Table S2).

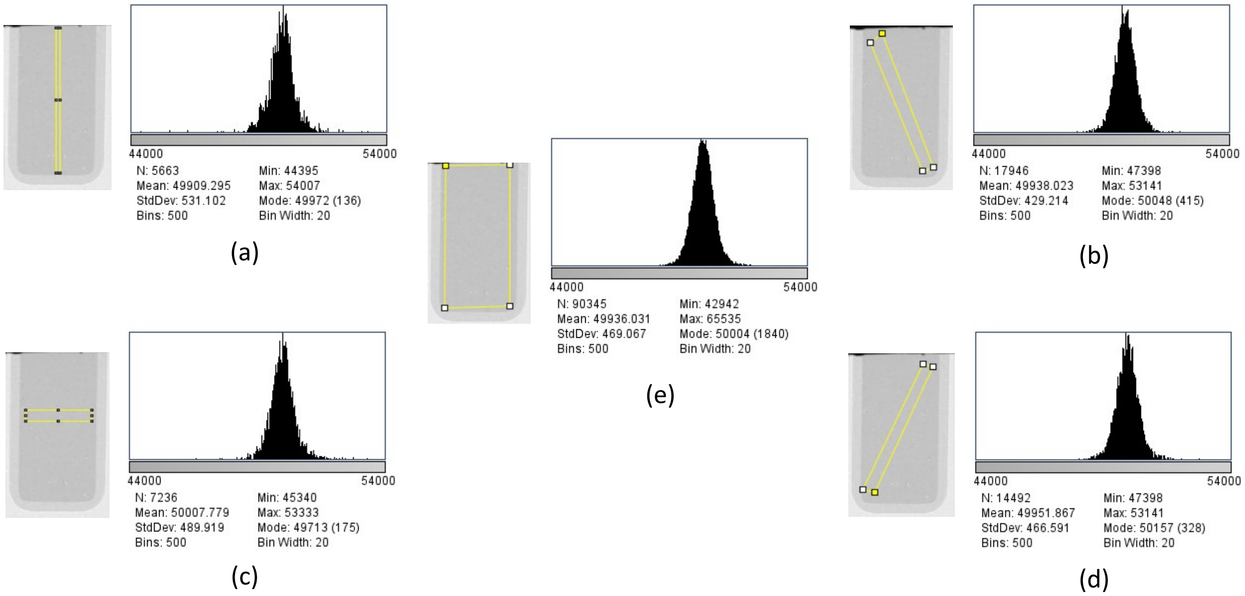

Figure S1. : Intensity histograms for different chosen ROIs within the same label. Figures **(a)-(d)** show thin ROI sections taken along four directions. Figure **(e)** shows entire ROI selected (See Figure S2 and Table S2, index 5).

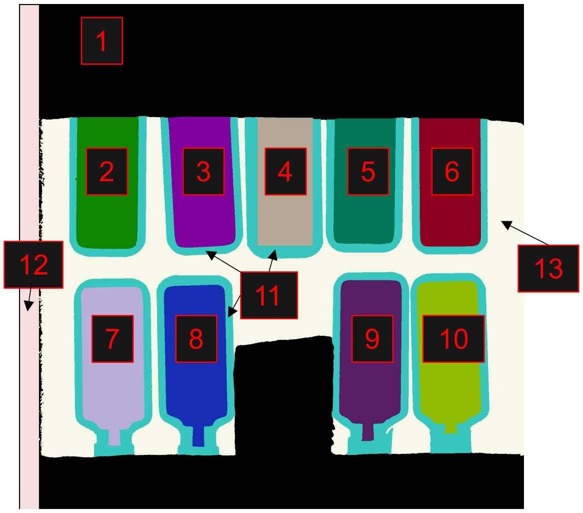

Figure S2. Colorized GT mask. Generated mask with look-up table colors applied to visually distinguish material class labels.

Index	Material
1	Dark background
2	PS Suspension (d=1000)
3	PS Suspension (d=500)
4	Control 1 - empty quartz
5	Control 2 - D_2O solution
6	PS Suspension (d=198)
7	PS Suspension (d=145)
8	PS Suspension (d=101)
9	PS Suspension (d=65)
10	PS Suspension (d=50)
11	Quartz holder
12	Side background
13	Air background

Table S2. Sample information. Indices associated with ROIs and material class labels corresponding to Figure S2. All diameter measurements are in nanometers.

3 Scene augmentation and checkerboard masks

As mentioned in the discussion section of the paper, we observe that using checkerboard patterns as scene simulations yielded higher accuracy of a trained AI model than using the original scene with spatial perturbations and material permutations as scene simulations (seen in FigureS3 as well as measured data). This is because the class characteristics are contained more along the autocorrelation and imaging modes axes of the data than along the spatial axes of the data. Therefore, training on augmented original scenes might encode a specific spatial layout as one of the key characteristics of classes in the trained AI model (i.e., the model will memorize the spatial layout). Such a trained model will then misclassify many pixels in images with

smaller ROIs than those present in the training data. In addition, augmented original scenes may not achieve class balancing while the checkerboard scenes will minimize the likelihood of class imbalance (see Figure S4).

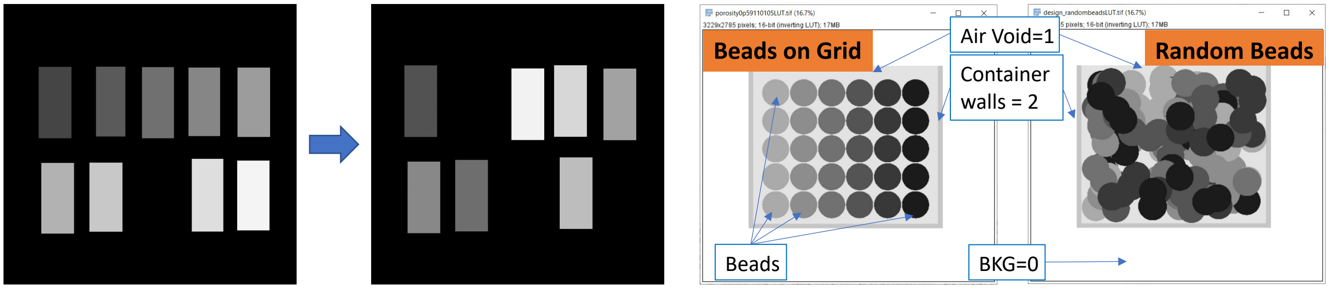

(a) (b)

Figure S3. Synthetic scene augmentations (a) Example of material/background permutations and location perturbations. Left – original simulated mask. Right – augmented mask. **(b)** Example of image masks based on future sample represented by a container with a grid of beads or randomly placed beads.

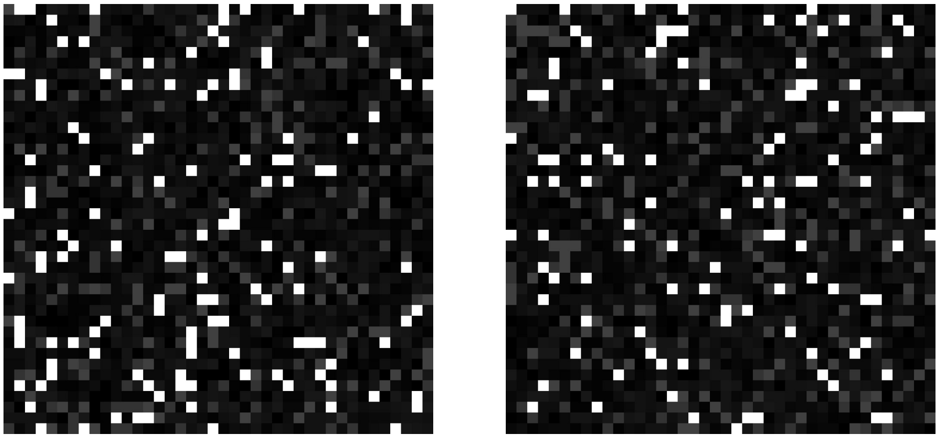

Figure S4. Examples of image mask design with a class balancing constraint. The checkerboard has checkers of size 50 pixels in the vertical and horizontal dimensions. Each checkerboard is a grid of 40 x 40 squares.

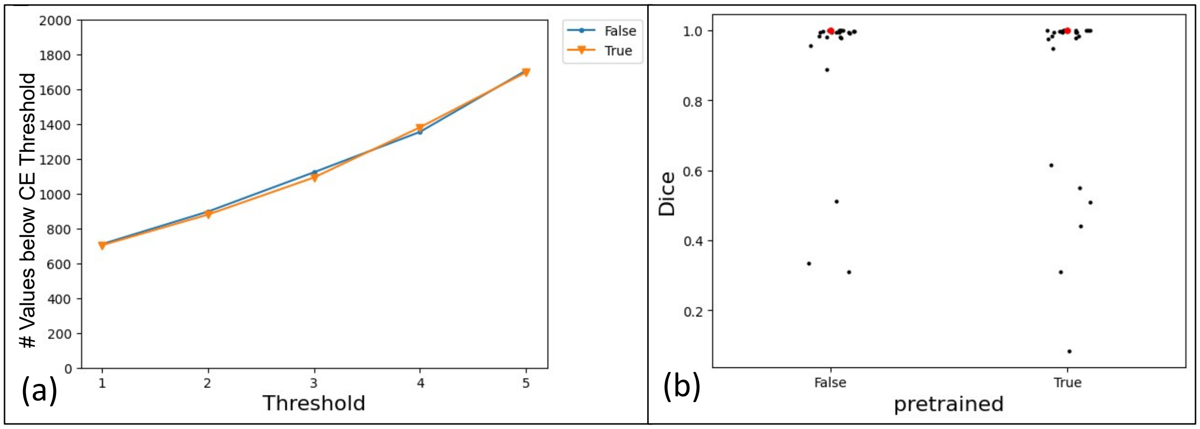

Figure S5. Impact of pretraining on model metrics. Model metrics - model convergence speed and Accuracy are compared for models with and without pretraining.

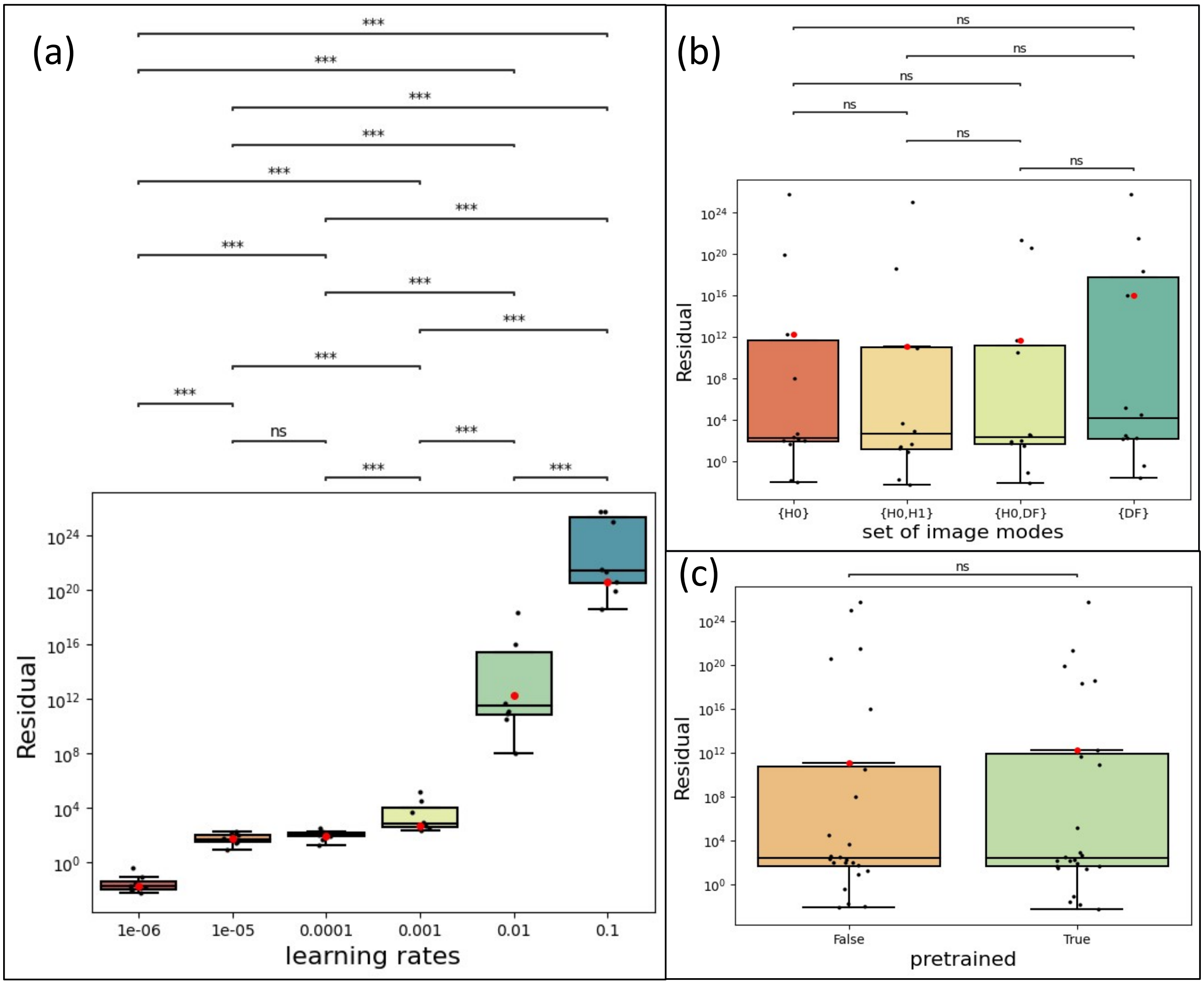

Figure S6. Effect of set of imaging modes, pretraining state and learning rates on model stability. Figure **(a)** shows the effect of learning rate, **(b)** set of imaging modes, and **(c)** pretraining on COCO dataset

4 Analyses of AI model training curves with respect to jitters and stability

Stability metrics showed that many of the tested models have jitters in their AI model training curves which could be due to normalization, batch size, and class imbalance in image tiles. Stability metrics may be considered to be inversely related to jitters as they are inversely related to residuals. As previously described, imaging mode sets and pretraining states do not show any significant impact on the models. These results can be seen in Figure S6. However, learning rates show a clear impact on the residual values, which are inversely related to model stability.

To illustrate results of the Mann-Whitney U test in S6, we use the star-notation to denote levels of statistical significance in our results. One asterisk (*) denotes a p-value of $p < 0.05$, and 2, 3, 4 asterisks denote p-values, respectively, of $p < 0.01$, $p < 0.001$, $p < 0.0001$. The abbreviation "ns" denotes that the differences are not statistically significant.

Jitters are noticed in all models with a learning rate above 10^{-4}. Adding batch normalization in Feature Extractor did not reduce these jitters in training curves (cross entropy as a function of epoch index). Based on our experiments, the optimal configuration was without batch normalization in Feature Extractor.

5 Scalability of the approach

In addition to plots using the Dice score as a metric, we also used Cross Entropy (CE) as a metric. The same conclusions can be drawn overall from the two metrics. However, the best model according to both metrics is not necessarily the same. Adjusted Rand index was also compared and it is usually reasonably close (few percent) to Dice coefficient. The dataset using imaging mode {H0} has fewer input 'channels' as compared to the dataset using imaging mode set {H0,H1} and therefore CE does not measure the same quantity. On the other hand, the Dice coefficient is independent of the number of input 'channels' and therefore a more objective method for comparing the results. Regardless of these differences, both approaches lead us to the same conclusion.

Additionally, in our experiments, we found no significant difference between using a DeepLab 50 model that was pretrained or not pretrained (randomly initialized). This can be seen in all the metrics derived from Train and Validation CE values as shown in Figure S5. The Residual/model stability metric can be seen in Figure S6(c). The results are the same when using the Dice score as a metric.

In the current work, we used a combination of the ImageJ/Fiji thresholding tool and manual painting of pixel labels to form a segmentation mask. The manual pixel painting can become a scalability bottleneck. The manual painting becomes labor demanding as (1) the number of measured images increases with the number of 2D tomographic projections and with the number of physical samples (currently it was one projection and one sample) and (2) the number of ROIs increases with the number of unique labels/materials and with the number of contiguous regions per label (currently 13 unique labels in 22 contiguous regions). As such, scaling the workflow to larger experimental setups will require designing semi-automated segmentation approaches and using a higher pixel bit depth for masks (currently 8 bits-per-pixels limiting the range to 255 labels).

6 Comparison to clustering

Clustering may be considered as a baseline method used to compare any novel method with. If we assume our reference materials to be homogeneous, the data to be a stack of registered 2D images as a function of autocorrelation, and manually established ROIs are available for reference materials over the stack of 2D images as a function of autocorrelation; then we can say that pixels from each ROI should have similar autocorrelation lengths and these are unique across all ROIs representing the reference materials. With the hypothesis that clustering of pixels from ROIs should yield one cluster per ROI.

For this we generated GPU-enabled k-means clustering with 13 clusters, treating each pixel in a 2D image as a point with 84 autocorrelation dimensions. Image mode DF was used as a baseline, since it is expected to have the most information in INFER data. From figure S7, we can observe that each ROI gets multiple cluster labels. Histograms of frequency of cluster labels per ROI were calculated and it was seen that only ROIs 5 and 9 satisfy the hypothesis. Potential sources identified for failure of this hypothesis are: (1) Heterogeneity of reference materials in addition to noise (2) Inaccurate registration of autocorrelation frames.

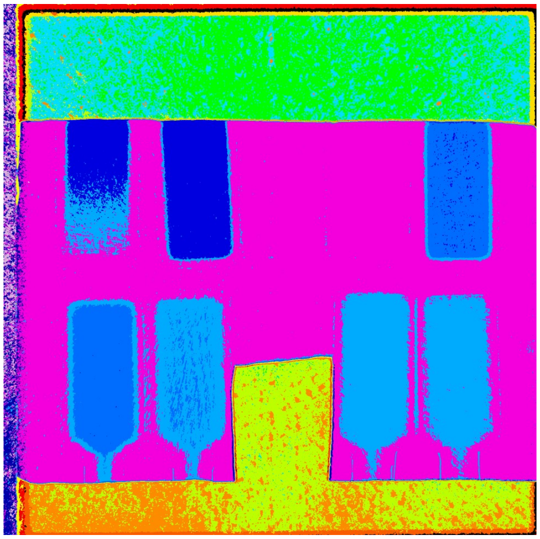

Figure S7. Kmeans clustering results for n=13 clusters after running for 281 iterations.

7 Creaming of polystyrene beads

While the expected masks for sample at Index 2 is a polystyrene suspension of diameter 1000 nm, when running test on the same sample, we observe layers. Figure S8 shows the analysis of this phenomenon. Figure S8(c), (b) respectively show the selected regions within the 1000 nm sample and its corresponding correlograms. FigureS8(d) shows the correlograms for each of the samples separately. As can be observed, while the correlograms in (b) do not have a 'knee', the ones in (d) have it. The 'knee' gives us information regarding the size of the particles based on the sphere model. Lack of change in knee but increasing slope in loss of visibility the higher you go in the sample indicate a changed concentration caused by more of the beads moving up and consequently having less at the bottom. We can explain the behaviour of the AI model as even though the actual correlograms are different, the models attempt to match the closest correlogram among the samples they are trained on. Creaming is observed based on the same physical principles as sedimentation.

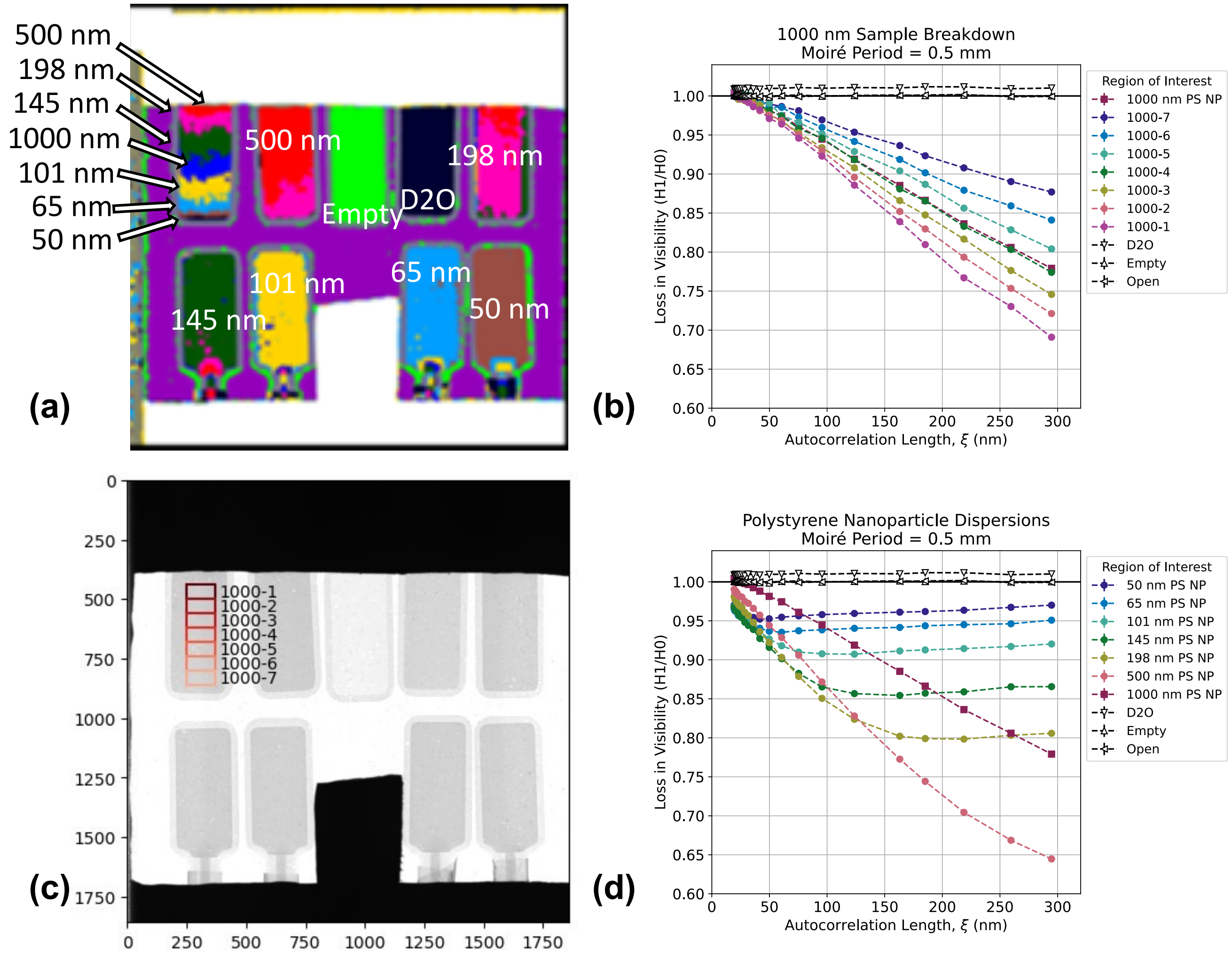

Figure S8. Observation and analysis of creaming effect in 1000 nm PS sample based on prediction. Figure **(a)** shows predicted labelled values with annotation. Figure **(b)** shows correlograms for each selected region in the 1000 nm ROI for smaller ROIs shown in Figure **(c)**. Figure **(d)** shows the correlograms for each sample

Based on the observation of creaming, we re-evaluated the assumption of a unimodal PDF in each GT region. We selected images for the first and last autocorrelation lengths based on Figure S8 and computed histograms along vertical and horizontal sub-regions shown in Figures S9 and S10. Since creaming causes a gradual shift of the PDF per layer, the PDF of the entire ROI is a sum of slightly shifted PDFs yielding a unimodal PDF. Thus, our statistical modelling assumption about unimodal distribution but the 1D PDF assumption is not for this ROI (Index 2). Other ROIs in the field of view contain intensity distributions deemed by the AI model to be closer to the intensity distributions of the layers in the ROI Index 2.

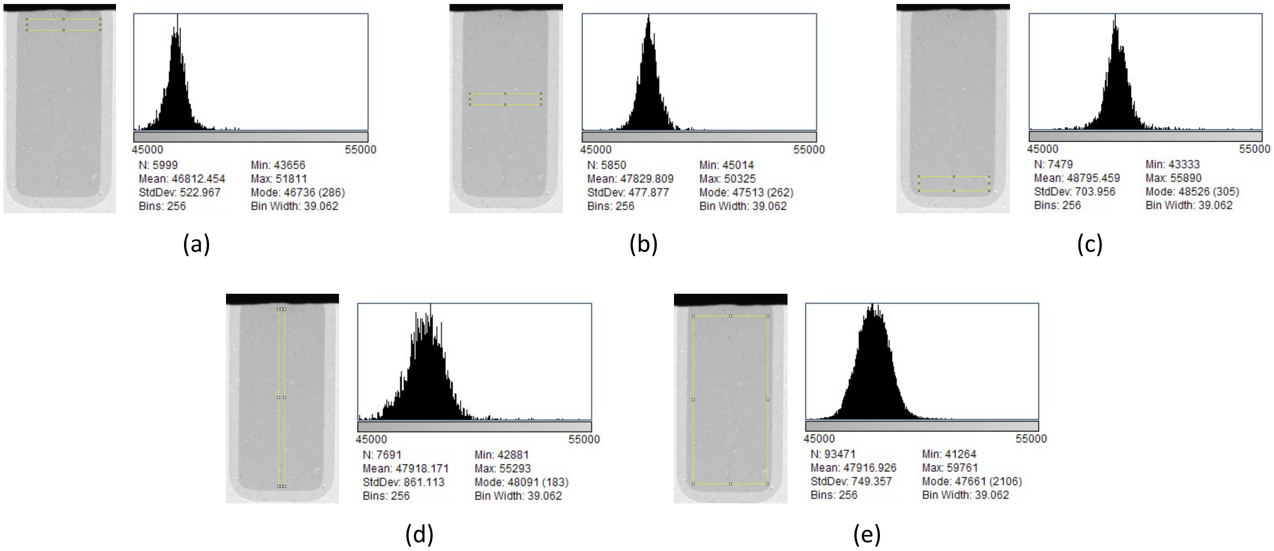

Figure S9. Histograms of ROI at index 2 at minimum autocorrelation length. Figures (a)-(c) show thin ROI sections along horizontal directions at different locations and Figure (d) shows thin ROI section along the vertical direction. Figure (e) shows entire ROI selected (See Figure S2 and Table S2, index 2)

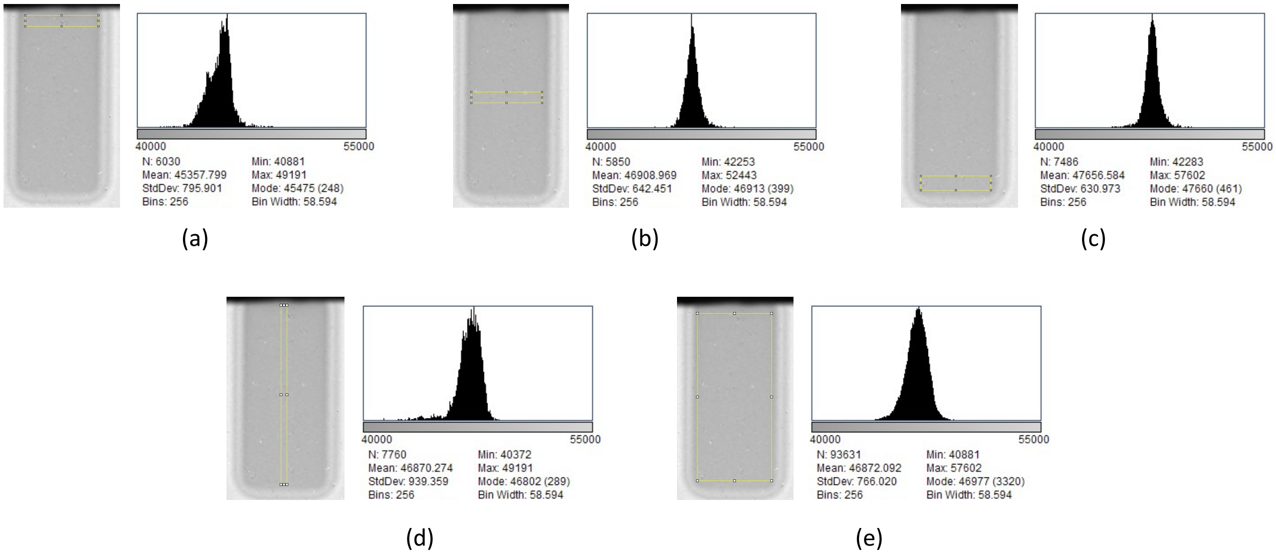

Figure S10. Histograms of ROI at index 2 at maximum autocorrelation length. Figures (a)-(c) show thin ROI sections along horizontal directions at different locations and Figure (d) shows thin ROI section along the vertical direction. Figure (e) shows entire ROI selected (See Figure S2 and Table S2, index 2)

8 Validation of the approach on a different measurement

To evaluate whether our approach will generalize to other samples, we applied it to a measurement of Amorphous Solid Dispersion (ASD) sample. This sample was also imaged with the CG-1D ORNL-HFIR instrument (see Section 6.1). This sample had 39 autocorrelations per image mode as opposed to 84 in the polystyrene sample (see section 2). Figure S11 and Table S3 show the GT mask and the sample specifications for the ASD sample. We followed the protocol described in Section 6.2.2 - Estimation, Design (Checkerboard mask), Generation of train, test and validation sets. Fifteen checkerboard masks were created for both train+validation and test sets according to 6.2.3. AI models were trained on both measured and synthetic data. The result are presented in Figure S12 and S4 over the hyperpameters : learning rates :$\{1e-3, 1e-4, 1e-5, 1e-6\} \times \{RandomInitialization\}$.The results from this measurement validate that the approach generalizes to another sample.

Similarly to the PS sample, S12 shows some layering of the segmentation results (middle row) with respect to the ASD sample GT mask. We explored the intensity distributions along those layers as shown in S13. This supports the prediction made by the model trained on Synthetic data. It should be noted that while physically these samples are known to have heterogeneities, these intensity variations are not visible to the eye, but the model trained on checkerboard data is able to capture them.

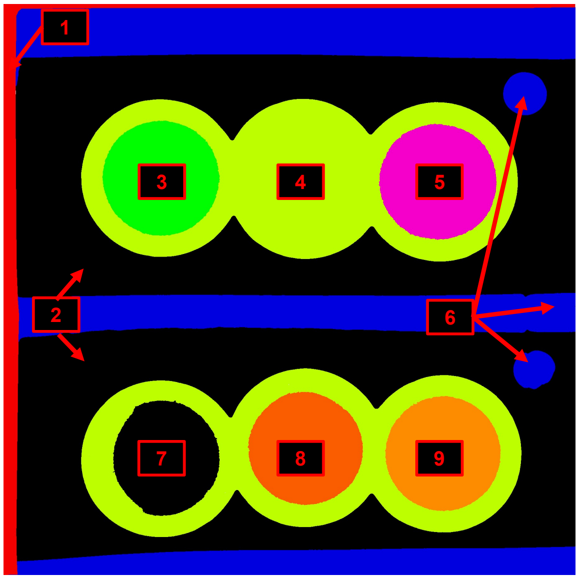

Figure S11. Colorized GT mask. Generated mask with look-up table colors applied to visually distinguish material class labels for ASD sample.

Index	Material
1	Background 1
2	Blocked Background
3	10% Palmitic-d31 Acid in PLGA - Acid Term.
4	Empty Cell
5	0% Palmitic-d31 Acid in PLGA - Acid Term.
6	Open region
7	10% Palmitic-d31 Acid in PLGA - Acid Term. - Annealed
8	25% Pamitic-d31 Acid in PLGA - Acid Term
9	10% Palmitic-d31 Acid in PLGA - Acid Term. - Annealed

Table S3. Sample information. Indices associated with ROIs and material class labels corresponding to Figure S11. PLGA is the poly (lactide-co-glycolide) matrix polymer and Acid term. stands for acid terminated groups on the polymer chains. All concentrations are in mass percentages.

Dice		Evaluate	
		Synthetic	Measured
Train	Synthetic	0.9849	0.7879
	Measured	0.3043	0.9380
**IoU score**		**Evaluate**	
		Synthetic	Measured
Train	Synthetic	0.9705	0.6925
	Measured	0.1803	0.9138

Table S4. Segmentation quality metrics. Comparison of Dice and IoU scores for the best (highest Dice score) model when trained on data-driven or measured and tested on data-driven or measured datasets. Best models selected using the highest Dice score.

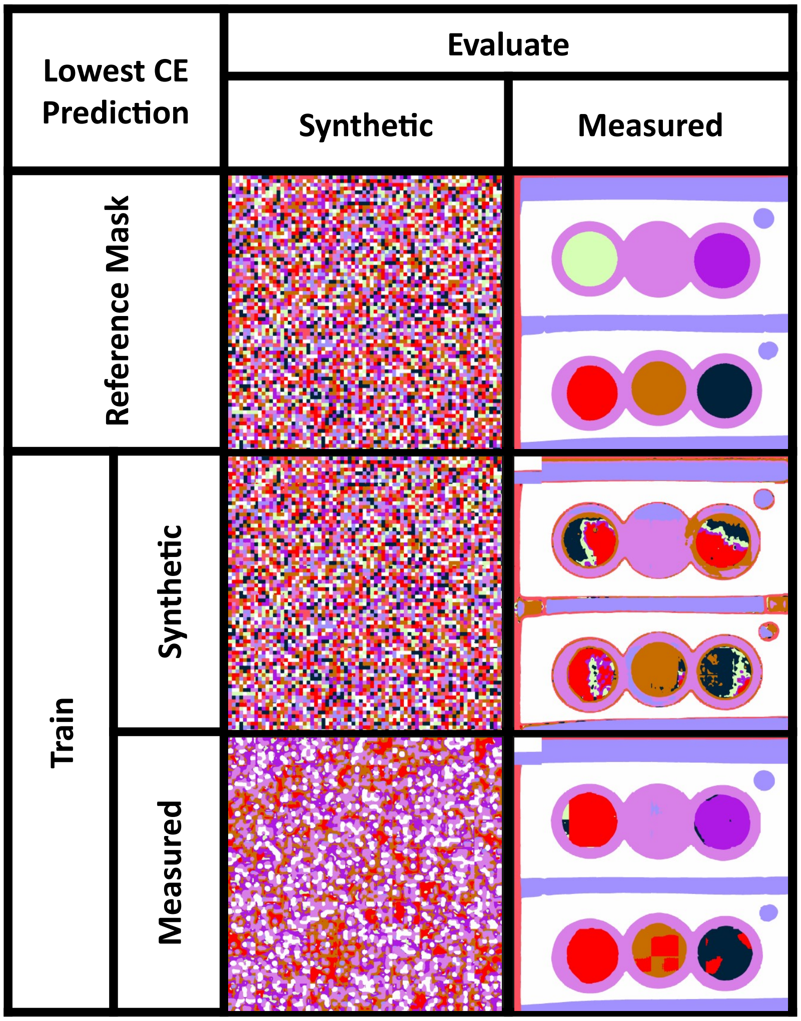

Figure S12. ASD sample: Train-Evaluation pairs for optimal models. Segmentation masks obtained with the most accurate AI model for each train/evaluate pair within tested hyperparameters. The row with the reference mask shows GT masks for synthetic and measured images. The four cells in the figure that are at the intersection of Train rows and Evaluate columns correspond to four combinations of training and evaluation to be compared with the row with the reference masks.

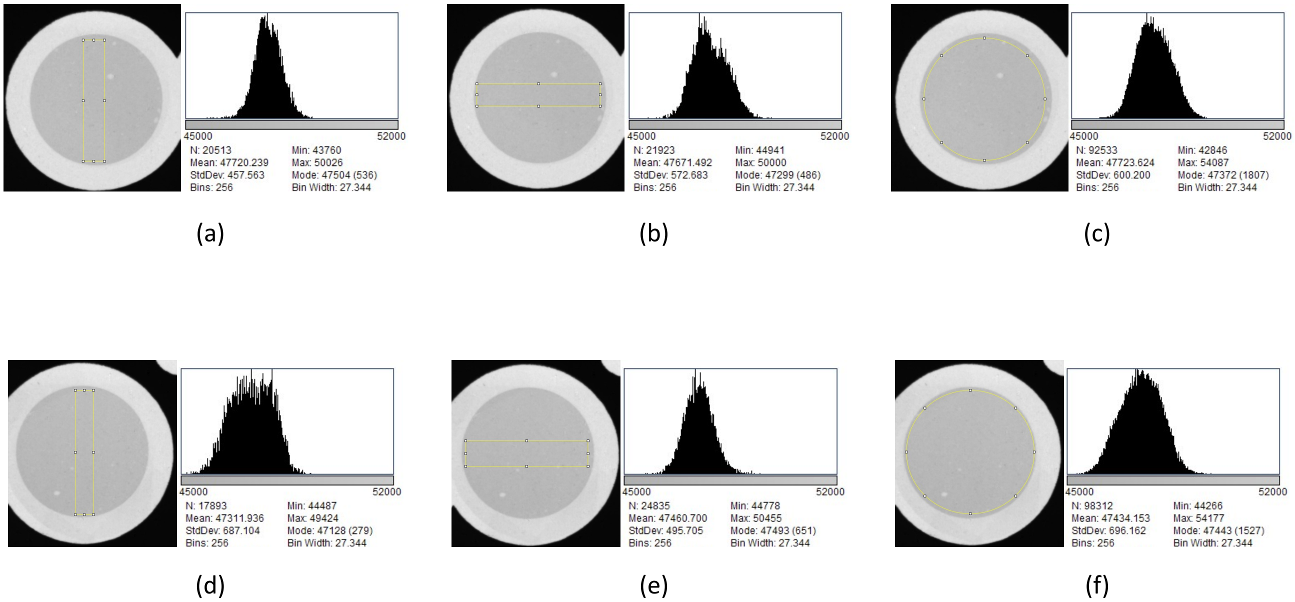

Figure S13. Histograms of ROI at indices 3 and 5 at minimum autocorrelation length. Figures (a)-(c) and (d)-(f) show ROI section along the vertical and horizontal directions, and entire region (See Figure S11 and Table S3, index 3 and index 5 respectively)

Supplementary figure Legends

Figure S1: : Intensity histograms for different chosen ROIs within the same label. Figures **(a)**-**(d)** show thin ROI sections taken along four directions. Figure **(e)** shows entire ROI selected (See Figure S2 and Table S2, index 5).

Figure S2: Colorized GT mask. Generated mask with look-up table colors applied to visually distinguish material class labels.

Figure S3: Synthetic scene augmentations (a) Example of material/background permutations and location perturbations. Left – original simulated mask. Right – augmented mask. **(b)** Example of image masks based on future sample represented by a container with a grid of beads or randomly placed beads.

Figure S4: Examples of image mask design with a class balancing constraint. The checkerboard has checkers of size 50 pixels in the vertical and horizontal dimensions. Each checkerboard is a grid of 40 x 40 squares.

Figure S5: Impact of pretraining on model metrics. Model metrics - model convergence speed and Accuracy are compared for models with and without pretraining.

Figure S6: Effect of set of imaging modes, pretraining state and learning rates on model stability. Figure **(a)** shows the effect of learning rate, **(b)** set of imaging modes, and **(c)** pretraining on COCO dataset

Figure S7: Kmeans clustering results for n=13 clusters after running for 281 iterations.

Figure S8: Observation and analysis of creaming effect in 1000 nm PS sample based on prediction. Figure **(a)** shows predicted labelled values with annotation. Figure **(b)** shows correlograms for each selected region in the 1000 nm ROI for smaller ROIs shown in Figure **(c)**. Figure **(d)** shows the correlograms for each sample

Figure S9: Histograms of ROI at index 2 at minimum autocorrelation length. Figures (a)-(c) show thin ROI sections along horizontal directions at different locations and Figure (d) shows thin ROI section along the vertical direction. Figure (e) shows entire ROI selected (See Figure S2 and Table S2, index 2)

Figure S10: Histograms of ROI at index 2 at maximum autocorrelation length. Figures (a)-(c) show thin ROI sections along horizontal directions at different locations and Figure (d) shows thin ROI section along the vertical direction. Figure (e) shows entire ROI selected (See Figure S2 and Table S2, index 2)

Figure S11: Colorized GT mask. Generated mask with look-up table colors applied to visually distinguish material class labels for ASD sample.

Figure S12: ASD sample: Train-Evaluation pairs for optimal models. Segmentation masks obtained with the most accurate AI model for each train/evaluate pair within tested hyperparameters. The row with the reference mask shows GT masks for synthetic and measured images. The four cells in the figure that are at the intersection of Train rows and Evaluate columns correspond to four combinations of training and evaluation to be compared with the row with the reference masks.

Figure S13: Histograms of ROI at indices 3 and 5 at minimum autocorrelation length. Figures (a)-(c) and (d)-(f) show ROI section along the vertical and horizontal directions, and entire region (See Figure S11 and Table S3, index 3 and index 5 respectively)

Supplementary table Legends

Table S1: Members of Johnson's family of probability density functions (PDFs)

Table S2: Sample information. Indices associated with ROIs and material class labels corresponding to Figure S2. All diameter measurements are in nanometers.

Table S3: Sample information. Indices associated with ROIs and material class labels corresponding to Figure S11. PLGA is the poly (lactide-co-glycolide) matrix polymer and Acid term. stands for acid terminated groups on the polymer chains. All concentrations are in mass percentages.

Table S4: Segmentation quality metrics. Comparison of Dice and IoU scores for the best (highest Dice score) model when trained on data-driven or measured and tested on data-driven or measured datasets. Best models selected using the highest Dice score.
